# Molecular and Functional Bases of Selection against a Mutation Bias in an RNA Virus

**DOI:** 10.1093/gbe/evx075

**Published:** 2017-05-01

**Authors:** Ignacio de la Higuera, Cristina Ferrer-Orta, Ana I. de Ávila, Celia Perales, Macarena Sierra, Kamalendra Singh, Stefan G. Sarafianos, Yves Dehouck, Ugo Bastolla, Nuria Verdaguer, Esteban Domingo

**Affiliations:** 1 Centro de Biología Molecular “Severo Ochoa” (CSIC-UAM), Consejo Superior de Investigaciones Científicas (CSIC), Campus de Cantoblanco, Madrid, Spain; 2 Christopher S. Bond Life Sciences Center and Department of Molecular Microbiology & Immunology, School of Medicine, University of Missouri, Columbia, Missouri; 3 Institut de Biologia Molecular de Barcelona (CSIC), Parc Científic de Barcelona, Barcelona, Spain; 4 Centro de Investigación Biomédica en Red de Enfermedades Hepáticas y Digestivas (CIBERehd), Barcelona, Spain; 5 Liver Unit, Internal Medicine, Laboratory of Malalties Hepàtiques, Vall d’Hebron Institut de Recerca-Hospital Universitari Vall d’Hebron (VHIR-HUVH), Universitat Autònoma de Barcelona, Barcelona, Spain; 6 Machine Learning Group, Université Libre de Bruxelles (ULB), Brussels, Belgium

**Keywords:** antiviral resistance, fitness, lethal mutagenesis, foot-and-mouth disease virus, 5-fluorouracil, protein folding stability

## Abstract

The selective pressures acting on viruses that replicate under enhanced mutation rates are largely unknown. Here, we describe resistance of foot-and-mouth disease virus to the mutagen 5-fluorouracil (FU) through a single polymerase substitution that prevents an excess of A to G and U to C transitions evoked by FU on the wild-type foot-and-mouth disease virus, while maintaining the same level of mutant spectrum complexity. The polymerase substitution inflicts upon the virus a fitness loss during replication in absence of FU but confers a fitness gain in presence of FU. The compensation of mutational bias was documented by in vitro nucleotide incorporation assays, and it was associated with structural modifications at the N-terminal region and motif B of the viral polymerase. Predictions of the effect of mutations that increase the frequency of G and C in the viral genome and encoded polymerase suggest multiple points in the virus life cycle where the mutational bias in favor of G and C may be detrimental. Application of predictive algorithms suggests adverse effects of the FU-directed mutational bias on protein stability. The results reinforce modulation of nucleotide incorporation as a lethal mutagenesis-escape mechanism (that permits eluding virus extinction despite replication in the presence of a mutagenic agent) and suggest that mutational bias can be a target of selection during virus replication.

## Introduction

RNA viruses exploit high mutation rates to generate complex mutant spectra (ensemble of genetic variants) which allow finding mutational pathways towards adaptation (Andino and Domingo 2015; Domingo 2016; Domingo et al. 1978; Lauring et al. 2013). Increasing the mutation rate above the basal level determined by the replication machinery can generate altered viral RNA and proteins in detriment of infectious progeny production. This is the basis of an antiviral strategy termed lethal mutagenesis in which virus-specific mutagenic agents such as nucleotide analogues increase mutation rates above a survival threshold (Andino and Domingo 2015; Dapp et al. 2013; Domingo 2005; Domingo and Schuster 2016a; Domingo et al. 2012; Graci and Cameron 2008; Holland et al. 1990; Loeb et al. 1999). However, viruses display molecular mechanisms to overcome the effect of mutagenic activities mediated by nucleotide analogues. When dependent on the polymerase, resistance can be achieved through a general increase of polymerase fidelity, a specific restriction to incorporate the mutagenic nucleotide, or modulation of nucleotide incorporation to achieve a balance among mutation types (Agudo et al. 2010; Borderia et al. 2016; Ferrer-Orta et al. 2010; Pfeiffer and Kirkegaard 2005; Vignuzzi et al. 2006; Zeng et al. 2013). Interestingly, modulation of mutation types in the picornavirus foot-and-mouth disease virus (FMDV) to confer resistance to the purine analogue ribavirin can be achieved not only through amino acid replacements in the viral polymerase (termed 3D; Agudo et al. 2010) but also by a single amino acid substitution in non-structural protein 2C, without the need of mutations in the polymerase (Agudo et al. 2016). Thus, modulation of nucleotide incorporation is a well established mechanism to escape ribavirin-induced lethal mutagenesis of FMDV.

The pyrimidine analogue 5-fluorouracil (FU) is mutagenic for several RNA viruses (Agudo et al. 2009; Holland et al. 1990), and replication in the presence of FU can lead to virus extinction in cell culture and in vivo (Grande-Pérez, Gómez-Mariano, et al. 2005; Grande-Pérez, Lázaro, et al. 2005; Grande-Pérez et al. 2002). FU is intracellularly converted into fluorouridine-triphosphate (FUTP), which is then incorporated as fluorouridine-monophosphate (FUMP) into RNA and can compete with incorporation of the standard nucleotides. FU exerts both an inhibitory and a mutagenic activity on FMDV (Agudo et al. 2008). As inhibitor, FU in its FUTP form blocks initiation of viral RNA synthesis. As mutagen, FUTP is incorporated during viral RNA elongation, and it evokes mainly A → G and U → C transitions. Here, we describe the selection, isolation and characterization of a FMDV mutant with substitution V173I in the F motif of 3D which is able to survive the mutagenic activity of FU. V173I does not produce direct resistance to the inhibition of FMDV replication by FU, but compensates for the increase of A → G and U → C transitions that the wild-type (wt) virus endures in the presence of FU. Compensation in the mutant virus entails an increase of G → A and C → U transitions in the presence of FU that approximates the mutational pattern to that of the wt virus replicating in the absence of FU. The same incorporation bias is observed with purified mutant polymerase using in vitro nucleotide incorporation assays. The structural modifications associated with the altered nucleotide incorporation have been examined by comparing the three-dimensional structures of the wt and mutant polymerases by X-ray crystallography. Structural changes have been identified at the N-terminal region of the protein. The codon composition of FMDV RNA predicts a higher frequency of non-synonymous mutations as a result of an increase of the proportion of G and C in the genome, with negative consequences for protein 3D stability. Experimental results and predictive algorithms of RNA and protein stability support that in the course of FU mutagenesis, a polymerase that can limit the frequency of A → G and U → C transitions will display a selective advantage over its wt counterpart. The results show that mutant spectrum composition can be a target of selection, and evoke mechanisms to escape lethal mutagenesis that do not involve a significant modification of polymerase fidelity or mutant spectrum complexity.

## Materials and Methods

### Cells, Viruses, and Infections

Baby hamster kidney 21 (BHK-21) cells were grown in Dulbecco’s modified Eagle’s medium (DMEM) (Gibco) supplemented with non-essential amino acids and 5% fetal calf serum, as previously described (Domingo et al. 1980). The origin of the FMDVs used in the present study is described in table 1. FMDV-RP [named Bp35 in Sierra et al. 2007] was subjected to 15 additional passages at a MOI of 0.05–0.1 plaque-forming units (PFU) per cell in the absence or presence of 50 μg/ml or 200 μg/ml of FU, leading to populations RP-15, RP(FU50)-15, and RP(FU200)-15, respectively. All infections were cytolytic and were allowed to progress until overt cytopathology (>80% cell killing), generally at 30–50 h post infection (p.i.). The procedures for infection of BHK-21 cell monolayers in liquid medium or under an agar overlay for titration of infectivity have been previously described (Domingo et al. 1980; Sobrino et al. 1983).

**Table 1:** Foot-and-Mouth Disease Viruses (FMDV) Used in the Present Study

Name	Origin	Reference
FMDV CS-8c1	Biological clone from natural isolate C_1_-Sta. Pau-Spain	Sobrino et al. 1983
pMT28	Molecular clone derived from CS-8c1	Toja et al. 1999
		García-Arriaza et al. 2005
FMDV MARLS	A monoclonal antibody-resistant mutant derived from FMDV C-S8c1 passaged 213 times in BHK-21 cells	Charpentier et al. 1996
FMDV-RP	A ribavirin-resistant mutant of FMDV MARLS^a^	Sierra et al. 2007

a FMDV MARLS displays high replicative fitness relative to FMDV C-S8c1, and this was the reason to use it as the parental FMDV population to derive the ribavirin-resistant mutant FMDV-RP.

### Treatment with 5-FU

FU was prepared in DMEM at a concentration of 2.5 mg/ml, and stored at −70 °C. Cells were preincubated with the desired concentration of FU for 7–13 h prior to infection; infections were allowed to proceed in the presence of the same drug concentration (Sierra et al. 2000).

### Preparation of FMDV with Substitution V173I

pMT28 (table 1) was used to construct plasmid pMT28-3D(V173I), encoding FMDV with amino acid substitution V173I in the polymerase (3D)-coding region. To this aim, pMT28 DNA was amplified with *PFU ultra* polymerase and primers with the desired nucleotide changes (the oligonucleotide primers used in the present study are listed in supplementary table S1, Supplementary Material online). A first amplification with V173Iplus and Pol1XbaI was followed by a second amplification with V173Iminus and PolCKpnI; the two distinct mutations in the relevant codon served as marker for the engineered virus. The products of both amplifications were shuffled and amplified with primers 5′3D and AV2New. Digestion of the DNA with BamHI (that cleaves at position 7427) and ClaI (position 7004) allowed ligation to pMT28 linearized with the same enzymes. Ligation, transformation of *Escherichia coli* DH5α, colony screening, nucleotide sequencing, transcription, and RNA transfections were carried out as previously described (Sierra et al. 2007). The effect of substitution V173I in 3D on FMDV replication was studied by comparing FMDV-wt with FMDV-3D(V173I), that differs from FMDV-wt only in the presence of mutations G7126A and A7128C (the first mutation leads to 3D with substitution V173I, and the second mutation serves as a marker for the construct). In this study we refer to FMDV-wt to mean a FMDV whose genomic sequence is identical to that of C-S8c1 or its molecular clone pMT28 (table 1). Likewise, wild type 3D refers to a polymerase 3D whose amino acid sequence is identical to the amino acid sequence of 3D encoded in C-S8c1 or its molecular clone pMT28.

### Fitness Assays

Relative fitness between FMDV-wt and FMDV-3D(V173I) was determined by growth-competition experiments in the absence or presence of FU, for a total of eight serial passages. The initial infection was carried out with a 1:1 ratio of infectivity of FMDV-wt:FMDV-3D(V173I). The proportion of the two viruses was determined by RT-PCR, by using discriminatory primers, as previously described (Agudo et al. 2010). The logarithm of the ratio of the two viruses was plotted against the passage number, and the fitness vector was adjusted to the exponential equation *y* = *a* . *e^bx^*; the antilogarithm of the vector slope gave the fitness value of FMDV-3D(V173I) relative to FMDV-wt. Control experiments showed that under the same passage conditions of the fitness assays, substitution V173I did not revert upon subjecting FMDV-3D(V173I) to 10 passages in absence of FU, and that FMDV-wt did not acquire substitution V173I after 10 passages in the presence of 400 μg/ml of FU.

### Quasispecies and Clonal Analyses

Quasispecies is the ensemble of related mutants subjected to a continuous process of genetic variation, competition among variants and selection of those that constitute a mutant spectrum at any given time. To sample the composition of the mutant spectrum generated in the viral populations and to obtain sequences for mutant polymerase preparation, cDNAs from single genomes (obtained from viral RNA through RT-PCR amplification with primers PolCKpnI and Pol1XbaI, spanning most of the 3D-coding region) were cloned into pGEM-T or pGEM-T Easy (Promega). To avoid redundant cloning of the same genome, an excess of viral RNA was ensured by using samples from which a 1/100 dilution produced a visible DNA band as the product of RT-PCR amplification (Airaksinen et al. 2003). *Escherichia coli* DH5α was transformed with the ligation products and DNA from individual positive colonies was amplified with Templiphi (GE Healthcare) and sequenced (Macrogen, Inc.).

### Computational Analyses

The Quickfold program on the DINAMelt server (http://unafold.rna.albany.edu/, Markham and Zuker 2005, 2008) was used to predict the secondary structure stability of the wild type RNA sequences encoding the 3D protein, and of the corresponding mutant RNA sequences observed in the cloned samples (supplementary tables S3 and S4, Supplementary Material online), or used to express the mutant 3D proteins analyzed by ThermoFluor. For each sequence, Quickfold predicted multiple structures with similar folding free energy, and we considered the lowest predicted free energy as an estimate of the RNA stability.

**Table 4:** Predicted RNA and Protein Stability, and Experimental Determination of Protein Stability of Several Mutant FMDV Polymerases (3D)

							Comments on 3D Purification		
Origin of FMDV Clone^a^	Mutations in 3D-Coding Region^b^	Amino Acid Substitutions in 3D^c^	Predicted RNA Stability ΔΔG^d^	Predicted Protein ΔΔG (kcal/mol), PoPMuSiC^e^	Predicted Protein ΔΔG /RT, DeltaGREM^f^	Experimental Tm ( °C) ThermoFluor^g^	Temperature Expression^h^	Yield (mg)^i^	Aggregation State (M, Monomer; D, Dimer, A, Aggregate)^j^
C-S8c1	None	None	0, reference	0, reference	0, reference	45.3 ± 1.0	37 °C	2.7	M, D
C-S8c1	A7291G, A7714G	T228A,K369E	−1.4	0.00	−0.09	45.0 ± 0.0	37 °C	2.9	M
Rib-resistant C-S8c1	C6739U, C7114U, G7497A	P44S, P169S, M296I	+3.0	1.73	−1.33	41.3 ± 0.6	37 °C	2.3	M, (D)
Rib-mutagenized C-S8c1	G6962A, G7324A, G7727A	G118D, V239M, G373D	+7.9	**4.63**	1.08	**34.0** ± **1.0**	37 °C	0.5	M, D
FU-resistant C-S8c1	G7126A, A7128C	V173I	−0.5	0.63	−0.27	44.6 ± 0.6	37 °C	3.0	M
FU-mutagenized C-S8c1	A6662G, A7195G	K18R, I196V	−1.7	1.20	−0.33	**36.3** ± **1.5**	20 °C	1.1	M
FU-mutagenized C-S8c1	U6671C, U6776C, G7126A, A7128C	L21P, I56T, V173I	+1.4	**5.48**	**5.52**	**32.3** ± **0.6**	37 °C	1.3	M, D, A
FU-mutagenized C-S8c1	A6689G, U6830C	H27R, F74S	−3.0	**3.70**	**7.13**	43.0 ± 0.0	20 °C	3.0	M
FU-mutagenized C-S8c1	U6773C, U7298C	V55A, F230S	+1.4	**4.69**	**8.95**	42.0 ± 1.0	37 °C	0.5	M, D, A
FU-mutagenized C-S8c1	A7274G, U7729C	D222G, F374L	−3.0	**2.54**	0.32	40.0 ± 0.0	20 °C	12	M
FU-mutagenized C-S8c1	G7126A, A7128C, G7336A, A7400G	V173I, A243T, E264G	−0.3	**2.44**	−0.42	N.A.	20 °C	N.D.	A

a C-S8c1 is the standard biological clone of FMDV. The 3D mutations tested were initially identified in 5-fluorouracil (FU)- or ribavirin (Rib)-resistant or mutagenized populations described in the present or previous studies (Arias et al. 2005; Agudo et al. 2010).

b The numbering of FMDV genomic residues is that described previously (Escarmis et al. 1999).

c The amino acid substitutions in 3D are indicated; numbering corresponds to the amino acids of 3D.

d Predictions based on Quickfold program on the DINAMelt server (see Materials and Methods).

e Change in 3D’s folding free energy resulting from the amino acid substitutions, as predicted by PoPMuSiC (see Materials and Methods). Values in bold indicate that an important alteration of 3D’s stability is predicted (ΔΔ*G* > 2.0 kcal/mol).

f Change in 3D’s folding free energy resulting from the amino acid substitutions, as predicted by DeltaGREM (see Materials and Methods). Values in bold indicate that an important alteration of 3D’s stability is predicted (ΔΔ*G*/RT > 2.0).

g The thermal stability of purified 3Ds (triplicate determinations, with standard deviation) were determined as described in Materials and Methods. Values in bold indicate an important alteration of 3D’s stability (*T*_m_ < 37 °C). N.A., not analyzed.

h The mutant 3Ds were expressed from plasmid pET-28a 3Dpol that included 3D with the amino acid substitutions given in the third column. Expression in *E. coli* was performed previously described (Ferrer-Orta et al. 2004) except that the temperature at which *E. coli* was grown had to be decreased to 20 °C to obtain 3D in sufficient yield and monomeric (M) form.

i The yield is expressed as the total amount of 3D (in mg) obtained from an *E. coli* culture of 500 ml grown as previously described (Ferrer-Orta et al. 2004).

j The forms observed are indicated. D in parenthesis in the triple P44S, P169I, M296I triple mutant means preponderance of the monomeric (M) form.

The impact of mutations on the folding free energy (ΔΔ*G*) of the 3D protein was computed using two different programs: PoPMuSiC and DeltaGREM. PoPMuSiC (http://babylone.ulb.ac.be/popmusic, Dehouck et al. 2011) relies on a set of statistical potentials derived from known protein structures, which describe the correlations between amino acid types, pairwise inter-residue distances, backbone torsion angles, and solvent accessibilities. It was shown to yield a correlation coefficient of 0.63 between predicted and experimental ΔΔ*G* values, in cross-validation on a set of 2,648 mutations in 131 different proteins (Dehouck et al. 2009). The changes in free energy resulting from substitutions in 3Dwt have been evaluated on the basis of seven different structures (PDB codes: 1U09, 1WNE, 2D7S, 2E9T, 2E9Z, 2EC0, 2F8E). The ΔΔ*G* predictions were very similar for all structures, and we report only the average values. In the case of 3D(V173I), the same structures were used (after introducing the V173I substitution), in addition to the two structures of 3D(V173I) presented here.

DeltaGREM (http://ub.cbm.uam.es/software/Delta_GREM.php) estimates the free energy of the native and the non-native state adopting the contact matrix representation of protein structures with the contact interaction parameters determined in Bastolla et al. (2000). The non-native state is modeled as the combination of the ensemble of unfolded structures, which dominates when the hydrophobicity is low, and the ensemble of compact, wrongly folded structures, which dominates when the hydrophobicity is high. The misfolded state is modeled through a Random Energy Model (Derrida 1981) of a large set of protein-like compact contact matrices, as described in Minning et al. (2013) [for computational details, see Arenas et al. 2015]. In the present study, the unfolded state was modeled through the configuration entropy of the protein chain. The free energy of a mutant was computed assuming that the native contact matrix does not change. This method yielded a correlation coefficient of 0.72 between predicted and experimental ΔΔ*G* for a set of 195 mutants that fold with two-state thermodynamics (Bastolla 2014).

For the DeltaGREM analysis, we used the available structures of the 3D protein (Agudo et al. 2010; Ferrer-Orta et al. 2010, 2004, 2015) as a template, adopting for each sequence the structural model that minimizes the predicted free energy, which in all cases corresponded to the structure with PDB code 1U09 (Agudo et al. 2010; Ferrer-Orta et al. 2004). Because all sequences generated by the mutant polymerase included also substitution V173I, we repeated the calculations considering mutant sequences where the substitution V173I had been computationally reverted; the mutation did not affect predicted stabilities. We also computed changes in hydrophobicity of the coded protein sequences, using the hydrophobicity scale derived by (Bastolla et al. 2005).

### Measurement of Polymerase Stability

The stability of wt and several mutant 3Ds that have been cloned, expressed and purified in the present study was compared using the ThermoFluor assay (Semisotnov et al. 1991), using a 96-well PCR plates (BIORAD). A total of 36 sample wells were prepared: three replicas of the 3Dwt, of the ten 3D mutants, and of the buffer control. Each well contained 42.5 μl of protein solution in 0.5M NaCl, 01.M Tris pH 8.5, 8% glycerol, 0.8 mM DTT and 0.8 mM EDTA buffer plus and 7.5 μl of diluted SYPRO Orange solution (ThermoFischer Scientific, S-6651) at 300× in the same buffer to reach a final volume of 50 μl and a final protein concentration of 1 mg/ml. The plates were then sealed with an adhesive optical clear seal (MicroAmp Optical Adhesive Film). Data were collected using IQ5 Multicolor Real-Time PCR Detection System (Biorad) and analyzed with iQ™5 Optical System Software. The fluorescence in each well was measured at regular intervals with a gradient of 0.5 °C per minute over a temperature range spanning from 30 to 95 °C. The interaction of the dye with the protein leads to a sigmoidal curve. The temperature at which 50% of the protein is unfolded and bound to the fluorescent dye (apparent melting temperature, *T*_m_) corresponds to the inflexion point of the slope. Raw data were plotted and analyzed using Excel-Microsoft.

### Preparation of FMDV Polymerases

To prepare the FMDV polymerase with amino acid substitution V173I, the 3D gene was cloned and expressed from vector pET-28a 3D*pol* [vector pET-28a (Novagen) with the 3D-coding region] using described procedures (Arias et al. 2005; Ferrer-Orta et al. 2004). 3D(V173I) was expressed from the corresponding mutant 3D constructed by site-directed mutagenesis with oligonucleotides V173Iplus and V173Iminus, that included nucleotidic changes G7126A and A7128C (supplementary table S1, Supplementary Material online), using the QuickChange site-directed mutagenesis kit (Stratagene). Enzyme expression and purification by affinity chromatography were performed as previously described (Agudo et al. 2010; Arias et al. 2005; Ferrer-Orta et al. 2004). Enzymes were >95% pure, according to analytical SDS-PAGE and Coomassie brilliant blue staining. The enzyme used for the crystallographic studies was subjected to an additional step of purification by size exclusion chromatography on a Superdex 200 HR column (GE Healthcare).

The same procedure was used to express and purify 3Ds containing two or three amino acid substitutions for studies of thermal stability. The polymerases were chosen among those encoded in genomes from the mutant spectra of FMDV populations passaged in the presence of FU or among ribavirin-resistant and ribavirin-mutagenized FMDVs mutants previously characterized in our laboratory; the aim was to test polymerases that span a broad range of predicted stability effects, their origin and amino acid sequence are given in Results. Temperature required for efficient expression in *E. coli*, protein yield and aggregation state were recorded for each polymerase.

### Polymerization Assays Using Heteropolymeric Symmetrical/Substrates (Sym/Sub) Template-Primers

Incorporation of standard nucleoside-5′-triphosphates or 5-fluorouridine-5′-triphosphate by the wt (3D) and mutant [3D(V173I)] polymerases was quantified using sym/sub RNAs (Dharmacon Research; Arnold and Cameron 2000; Castro et al. 2005; Gohara et al. 2000). The sym/sub RNAs are named according to the two nucleotides nearest to the duplex region; their sequence is given for each experiment. The oligonucleotides were purified, 5′-^32^P-end-labeled with [γ-P^32^] ATP by polynucleotide kinase (New England Biolabs), purified and annealed in 500 mM Tris–HCl (pH 7.8) and 1 M KCl, by heating at 95 °C for 5 min and slow cooling to room temperature. Nucleotide incorporation by 3D was monitored by incubating sym/sub RNA with 1 µM 3D (concentration as active site equivalents, measured by rapid quench flow, described below) and increasing concentration of the required NTP substrates for different time periods (Agudo et al. 2010; Arias et al. 2008). Polymerization products were resolved by electrophoresis on a denaturing 23% polyacrylamide, 7 M urea gel in 90 mM Tris-base, 90 mM boric acid, 2 mM EDTA, scanned with a Phosphorimager (BAS-1500; Fuji), and quantitated with ImageJ 1.45s (NIH).

### Rapid Quenched-Flow Assays

To measure incorporation of cognate nucleotides under presteady-state conditions, a model RQF-3 chemical quench-flow apparatus (KinTek Corp., Austin, TX) was used. Experiments were performed at 37 °C in 50 mM HEPES (pH 7.5), 5 mM MgCl_2_ and 10 mM 2-mercaptoethanol. Protein 3D was preincubated with the RNA template/primer and the first incoming NTP (10 µM) to allow formation of 3D-RNA_*n*+1_ product complexes. This reaction mixture was loaded in syringe A (sample loop volume 17.2 µl), and mixed with varying concentrations of NTP loaded in syringe B (sample loop volume 17.4 µl). Reactions were allowed to proceed and stopped with 0.5 M EDTA at different time periods (0.01–5 s). Reactions with each NTP concentration were tested for >6 time points. Products were resolved on a 23% polyacrylamide 7 M urea gel, and scanned with Typhoon FLA 9000 (GE Healthcare). The reaction products were quantitated with ImageJ 1.45s. Data were fitted by non-linear regression to the exponential equation *P* = *A*(1 – *e*^–*k*^^o^^bs^^*t*^) (where *A* is the amplitude of the burst, *k*_obs_ is the observed burst rate constant for NTP incorporation, and *t* is the reaction time), using the program GraphPad Prism 4 (GraphPad Inc.).

To obtain the dissociation constant *K*_d.NTP_ for NTP binding to the 3D-sym/sub complex, the observed burst rates (*k*_obs_) were plotted as a function of NTP concentration and the data were fitted by non-linear regression to the hyperbolic equation *k*_obs_ = (*k*_pol_[NTP])/(*K*_D-NTP_ + [NTP]) (where *k*_pol_ is the maximal rate for nucleotide incorporation) using GraphPad Prism 4.

To determine the proportion of active sites in the polymerase preparations, incorporation rates in 0.25 s were determined for 1 µM 3D in the presence of varying concentrations of RNA (0.1, 0.2, 0.5, 1, and 2 µM) and saturating NTP concentration (2 mM). Values of total amount of RNA elongated were plotted and fitted to a quadratic equation to obtain the concentration of active enzymes.

### Other Assays with 3D

The VPg uridylylation activity was measured employing two different protocols that include either Mn^2+^ and poly(A), or Mg^2+^ and cre as template, plus 3CD. In the assay in the presence of Mn^2+^ the reaction mixture contained 30 mM MOPS, pH 7.0, 33 mM NaCl, 0.6 mM MnCl_2_, 40 ng/µl poly(A) (average length of 300 residues), 150 µM VPg, 8% glycerol, 0.4 mg/ml bovine serum albumin (New England Biolabs), and 50 µM [α-^32^P UTP] (0.01 mCi/ml; 200 mCi/nmol) in the presence of different concentrations of FUTP. The reaction was carried out for 30 min at 37 °C, and it was stopped by adding EDTA to a final concentration of 83 mM. Relative activity was measured from the amount of VPg-pU(pU) determined electrophoretically as previously described (Agudo et al. 2008). For the assay in the presence of Mg^2+^ the reaction mixture contained 30 mM MOPS, pH 7.0, 33 mM NaCl, 5 mM MgCl_2_, 20 nM cre, 0.16 µM 3CD, 150 µM VPg, 8% glycerol, 0.4 mg/ml bovine serum albumin, and 5 µM [α-^32^P UTP] (0.01 mCi/ml; 200 mCi/nmol).

Binding of 3D to RNA was quantified by a gel mobility shift assay as previously described (Arias et al. 2005). The RNA binding mixture included 285 nM of the labeled sym/sub-UG, 100 mM MOPS (pH 7.0), 10 mM Mg(CH_3_COO)_2_, 5% polyethylene glycol, and increasing concentrations (0, 250, 500, 1,000, and 2,000 nM) of 3D. The mixtures were loaded onto a non-denaturing 4% polyacrylamide gel in TAE retarding buffer (120 mM Tris–acetate, 7 mM EDTA, pH 7.5) and 5% glycerol and electrophoresed at 200 V, 4 °C for 1 h in TAE retarding buffer; then the gel was fixed, dried and the free and complexed RNA was measured in a Phosphorimager (Arias et al. 2005).

### Crystallization and Structure Solution of 3D(V173I)

Purified 3D(V173I) was stored in a buffer containing Tris–HCl (50 mM pH 8.0), NaCl (500 mM), DTT (0.8 mM), EDTA (0.8 mM), and glycerol (8%), at a concentration of 5 mg/ml. Prior to the crystallization experiments, the polymerase was incubated overnight at 4 °C with oligonucleotide 5′ACGFuGGGCCC 3′ (NWG-Biotech) (oligonucleotide with FU-monophosphate at position 4), in an equimolar proportion, in presence of 2 mM MgCl_2_. Crystals were obtained at 20 °C by the hanging drop vapor diffusion method in limbro plates, using a precipitant/well solution containing 36% PEG 4000, 0.2 M ammonium acetate, 0.1 M MES[2-(N-morpholino) ethanesulfonic acid] pH 6.0, and 4% γ-butyrolactone. Two distinct crystal forms, which were suitable for X-ray analysis, were visible between 4 and 5 days after solution preparation. In order to obtain the ternary complexes, crystals were soaked for 6 h in a harvesting solution containing the crystallization buffer, 20 mM ATP and 5 mM MnCl_2_. Crystals were then transferred to a cryo-protecting solution, containing 20% glycerol in the crystallization buffer immediately before flash freezing in liquid nitrogen.

Two different data sets were collected at 100 K, using synchrotron radiation at ALBA-CELLS, beamline XALOC on a Pilatus 6M DECTRIS detector (*λ* = 0.979 Å). Trigonal crystals, belonging to the space group P3_2_21 diffracted to 2.4 Å resolution and tetragonal crystals, space group P4_1_2_1_2, diffracted to 2.6 Å resolution. All data were processed with XDS (Kabsch 2010) and internally scaled with SCALA [CCP4i, Potterton et al. 2003]. Data collection statistics are given in supplementary table S2, Supplementary Material online.

The initial maps for the tetragonal crystal structures were obtained after rigid-body fitting of the coordinates of the isolated wt polymerase [crystallized in the tetragonal P4_1_2_1_2 (PDB id. 1U09)] to the new unit cells, using the program Refmac5 [CCP4i, Murshudov et al. 1997]. Initial maps for the trigonal crystal form were obtained following the same procedure but using the P3_2_21 coordinates of 3Dwt (PDB id. 1WNE) as starting model (supplementary table S2, Supplementary Material online). In both structures, the weighted 2|Fo| – |Fc| and |Fo| – |Fc| difference maps clearly allowed the rebuilding of the mutated residue and other regions presenting conformational changes as a consequence of the mutation. Several cycles of automatic refinement, performed with Refmac5 (Murshudov et al. 1997), were alternated with manual model rebuilding using Coot (Emsley et al. 2010). The refinement statistics are summarized in supplementary table S2, Supplementary Material online.

## Results

### Selection of a FMDV Mutant Displaying Increased Fitness in the Presence of 5-FU

**Fig. 1.—:**
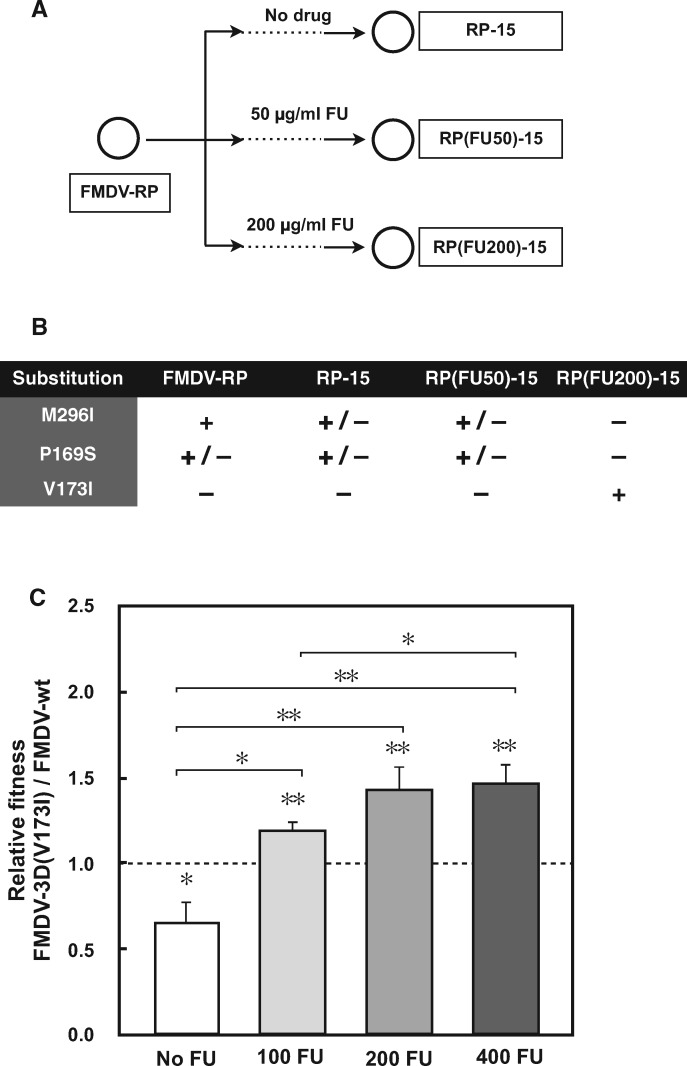
Passage history, acquisition of mutations in the polymerase (3D)-coding region, and fitness of FMDV-3D(V173I) relative to FMDV-wt in the presence of different FU concentrations. (*A*) FMDV-RP (its origin is described in Materials and Methods) was subjected to 15 passages in the absence or presence of 5-fluorouracil (FU) (50 µg/ml or 200 µg/ml added to culture medium) to yield populations RP-15, RP(FU50)-15 and RP(FU200)-15, respectively. (*B*) Amino acid substitutions in 3D of populations depicted in *A*, deduced from the corresponding consensus sequence of the 3Dcoding region; +, presence of mutation; −, absence of mutation; +/ −, presence of mutation in ∼50% of the RNA population, according to the corresponding nucleotide peak in the sequence. Numbering of genomic and amino acid residues is according to Escarmís et al. (1999). (*C*) To determine the relative fitness of FMDV-3D(V173I) and FMDV-wt, BHK-21 cells were infected with a mixture of FMDV-3D(V173I) and FMDV-wt; the RNA ratio of the two viruses ranged between 1:1 and 0.1:1, at a total initial MOI of 0.1 PFU/cell. Passages were performed in the presence of the concentration of FU (µg/ml) indicated below each bar. RNA of the two competing viruses was measured in triplicate. Fitness values were calculated as described in Materials and Methods; the fitness determination plots are given in supplementary figure S2, Supplementary Material online. The error bars indicate the error of the fit of each individual fitness value. Statistical significances are computed through the *t*-test, to evaluate if the slope of the ratio of RNA of the two competing viruses versus the passage number (nine points) is different from zero; significances are represented as (*P < 0.05; **P < 0.01). The statistical significance of the difference between pairs of fitness values is indicated on the lines linking two bars (*P < 0.05; **P < 0.005; Welch’s two-tailed test); *P* values above 0.05 were obtained in the comparison of fitness values obtained with 400 µM FU versus 200 µM FU, and 200 µM FU versus 100 µM FU. Procedures are further detailed in Materials and Methods.

FMDV-RP is a ribavirin-resistant population whose mutant spectrum included mutations that led to amino acid replacements M296I and P169S in the polymerase (3D) (at 100% and 50% dominance, respectively; Agudo et al. 2010; Sierra et al. 2007). To investigate a possible cross-resistance to FU, FMDV-RP was subjected to 15 passages in the absence or presence of FU (50 or 200 μg/ml) at a MOI of 0.05–0.1 PFU/cell (fig. 1*A*). Sequencing of the 3D-coding region indicated dominance (presence without alternative residues), at passage 15 in the presence of 200 μg/ml FU, of a new 3D substitution, V173I in the polymerase motif F (resulting from transition G7126A in the 3D-coding region), and loss of replacements M296I and P169S (fig. 1*B*). To determine if V173I alone conferred FU resistance, FMDV-3D(V173I) was prepared as described in Materials and Methods, and its response to FU compared with the response of FMDV-wt. FU displays a dual mutagenic and inhibitory activity on FMDV (Agudo et al. 2008) but FMDV-3D(V173I) did not exhibit a significant resistance to the inhibitory effect of FU (supplementary fig. S1, Supplementary Material online). The lack of effect on the inhibition by FU establishes a distinction between polymerase residues that can affect inhibition by FU and those that can affect its mutagenic properties. Growth-competition fitness assays indicated a fitness cost inflicted by replacement V173I in 3D in the absence of FU but a FU concentration-dependent fitness gain in the presence of FU (fig. 1*C* and supplementary fig. S2, Supplementary Material online); the selective strength (fitness_+drug_/fitness_-drug_) was 2.23 for substitution V173I with the maximum concentration of FU tested (400 µg/ml). Thus, 3D replacement V173I in FMDV 3D confers a selective advantage to the virus when it replicates in the presence of FU.

### Mutation Types and Mutant Spectrum Complexity of wt and Mutant Virus

We investigated how replacement V173I in 3D might affect quasispecies complexity in response to FU. Comparative analyses of the mutant spectra showed that passages in the presence of FU increased the mutation frequency for both FMDV-wt and FMDV-3D(V173I) (table 2; Pearson’s chi-squared test: *P* < 0.0001), resulting in a larger heterogeneity of the sequences in the mutant spectra. Comparison of mutation types and predicted amino acid substitutions indicated a predominance of transitions and non-synonymous mutations (supplementary tables S3 and S4, Supplementary Material online). The proportion of amino acid replacements with negative PAM 250 “(point accepted mutation matrix)” values ranged from 8% to 26% suggesting a good average acceptability of the amino acid substitutions in the polymerases of the viruses sampled from the mutant spectrum of the 3D-coding region; amino acid substitutions were found along the 3D-coding region (supplementary fig. S3, Supplementary Material online). The most salient difference between the two viruses regarding the mutation types was the proportion of A → G plus U → C relative to all mutations scored. For FMDV-wt such proportion was 0.67 in the mutant spectrum of the virus passaged 10 times in the absence of FU, and 0.84 in the virus passaged in the presence of FU (*P* = 0.04; Fisher’s test), a bias diagnostic of FU mutagenesis (Agudo et al. 2008; Grande-Pérez et al. 2002). Instead of an increase, a decrease of the mutational bias was observed in the mutant spectrum of mutant FMDV-3D(V173I): 0.76 in the absence of FU versus 0.73 in its presence (*P* > 0.05; Fisher’s test). The different response of the two viruses to FU can be expressed by the mutation type ratio normalized to the frequency of each nucleotide (named transition bias in table 2). The transition bias in the presence of FU increased 1.9-fold for FMDV-wt, and decreased 1.4-fold for FMDV-3D(V173I). Thus, the analyses suggest that the mutant polymerase tends to counteract the mutational bias toward A → G and U → C produced by FU mutagenesis.

**Table 2:** Quasispecies Analysis of the 3D-Coding Region of FMDV Populations Passaged in the Absence or Presence of 5-Fluorouracil^a^

						Synonymous Mutations (%)^g^	
Viral Population^a^	Number of Nucleotides (clones)^b^	Mutation Frequency [min]^c^	Mutation Frequency [max]^d^	Shannon Entropy^e^	Transition Bias^f^	Found (Expected) [min]^h^	Found (Expected) [max]^i^
FMDV-wt	72,610 (53)	5.9 × 10^−4^ (43)	6.2 × 10^−4^ (45)	0.7	3.3	34.9 (27.4)	35.6 (27.4)
FMDV-wt + FU	52,060 (38)	1.8 × 10^−3^ (96)	2.6 × 10^−3^ (137)	1.0	6.2	38.5* (29.5)	44.5** (29.2)
FMDV-3D(V173I)	68,500 (50)	4.8 × 10^−4^ (33)	5.0 × 10^−4^ (34)	0.5	4.7	18.2 (29.0)	17.6 (29.2)
FMDV-3D(V173I) + FU	58,910 (43)	1.7 × 10^−3^ (103)	2.0 × 10^−3^ (120)	1.0	3.3	43.7** (31.0)	47.5** (31.2)

a Wild-type FMDV or mutant FMDV-3D(V173I) were subjected to 10 serial passages in the absence or presence of FU (400 µg/ml) (+FU).

b The mutant spectrum analysis involved nucleotides 6,610–7,980 in the 3D-coding region; the number of individual clones analyzed is given in parenthesis.

c Mutation frequency [min] is the number of different mutations found in the mutant spectrum (relative to the sequence of the parental, unpassaged FMDV clone), divided by the total number of nucleotides sequenced (given in the second column); values in parenthesis are the total number of unique mutations. The statistical significance of the differences between values is the following: FMDV-wt, no FU versus FU: *χ*^2^ = 42.7, *P* = 6.5 × 10^−11^; FMDV-3D(V173I), no FU versus FU: *χ*^2^ = 47.6, *P* = 5.0 × 10^−12^; no FU, FMDV-wt versus FMDV-3D(V173I): *χ*^2^ = 0.79, *P* = 0.37; FU, FMDV-wt versus FMDV-3D(V173I): *χ*^2^ = 0.14, *P* = 0.71. (In all cases, Pearson’s chi-squared test, df = 1).

d Mutation frequency [max] is the number of total mutations found in the mutant spectrum (relative to the sequence of the parental, unpassaged FMDV clone), divided by the total number of nucleotides sequenced (given in the second column); values in parenthesis are the total number of mutations. The statistical significance of the differences between values is the following: FMDV-wt, no FU versus FU: *χ*^2^ = 84.1, *P* = 4.5 × 10^−12^; FMDV-3D(V173I), no FU versus FU: *χ*^2^ = 62.2, *P* = 2.9 × 10^−15^; no FU, FMDV-wt versus FMDV-3D(V173I): *χ*^2^ = 0.95, *P* = 0.33; FU, FMDV-wt versus FMDV-3D(V173I): *χ*^2^ = 4.2, *P* = 3.9 × 10^−2^. (In all cases, Pearson’s chi-squared test, df = 1).

e Shannon entropy (*S*) is calculated by the formula *S* =  − [*S_i_* (*p_i_* × ln *p_i_*)]/ln *N*, in which *p_i_* is the frequency of each sequence in the quasispecies, and *N* is the total number of sequences compared. It is a measure of heterogeneity of the sequences sampled in the mutant spectrum (*S* = 0, all sequences are identical; *S* = 1, each sequence is different).

f Transition bias is defined here as the ratio between the frequency of transitions A → G plus U → C, relative to the frequency of transitions G–A plus C–U: $\left[\left(\frac{A\to G}{A}\right)+\left(\frac{U\to C}{U}\right)/\left(\frac{G\to A}{G}\right)+\left(\frac{C\to U}{C}\right)\right]$.

g Actual and expected (in parenthesis) percentage of synonymous mutations. The expected values were computed from the sequence of the 3D-coding region of FMDV, assuming the all mutations of a given type are equally probable, and considering the same relative frequencies of different types of mutations as in the corresponding mutant spectra. Asterisks indicate a significant difference between the actual and expected values (*P < 0.05; **P < 0.01; binomial test). (See also footnotes h, i.)

h The statistical significance of the differences between the found and expected values is the following: FMDV-wt, no FU: *P* = 0.17; FMDV-wt, FU: *P* = 0.036; FMDV-3D(V173I), no FU: *P* = 0.94; FMDV-3D(V173I), FU: *P* = 0.004. (In all cases, Exact binomial test, df =1).

i The statistical significance of the differences between the found and expected values is the following: FMDV-wt, no FU: *P* = 0.1451; FMDV-wt, FU: *P* = 0.0001; FMDV-3D(V173I), no FU: *P* = 0.95; FMDV-3D(V173I), FU: *P* = 0.0001. (In all cases, Exact binomial test, df =1).

### Kinetics of Nucleotide Incorporation by wt and Mutant Polymerase

**Fig. 2.—:**
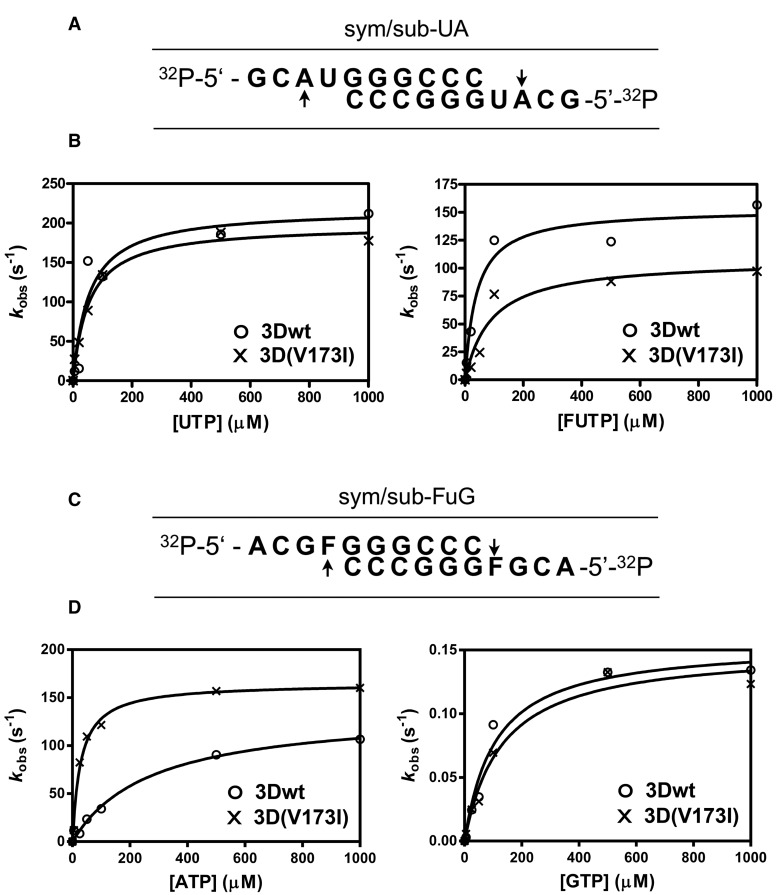
Presteady-state kinetics of nucleotide incorporation into sym/sub-UA and sym/sub-FuG by 3Dwt and 3D(V173I). (*A*) Sequence of 5′-end-labeled, annealed sym/sub-UA. Arrows indicate the template residue at which nucleotide incorporation is measured. (*B*) 3D (0.5 µM active sites) was preincubated at 37 °C with sym/sub-UA (0.5 µM duplex) and ATP (10 µM) for 900 s to allow the formation of 3D-RNA product complex, with AMP incorporated at the first position. The 3D-RNA product was then mixed with the indicated concentration of UTP or FUTP using a rapid chemical quench-flow apparatus, and reactions were quenched by the addition of EDTA (0.3 M). Independent time points at 0, 0.01, 0.05, 0.1, 0.25, 0.5, 1, and 2 s were taken for each nucleotide concentration tested. Time courses at fixed nucleotide concentrations were fit to an exponential curve to obtain the observed rate constant for nucleotide incorporation at the second position, *k*_obs_. The observed rate constants were then plotted as a function of nucleotide concentration, and the data were fit to a hyperbola to obtain *k*_pol_ and *K*_d,app_. Note that the scale at ordinate is different in the two panels. (*C*) Sequence of 5′-end-labeled, annealed sym/sub-FuG (Fu means FU). Arrows indicate the template residue at which nucleotide incorporation is measured (FU is written F). (*D*) Incorporation of AMP or GMP; no nucleotide was preincubated with 3D and RNA. Procedures are those described in (*B*). For the assays with GTP, the concentration of 3D used was 1 µM active sites, and no rapid chemical quench-flow apparatus was needed. Duplicate samples at 0, 10, 30, 60, 120, 300 and 900 s time points were taken for each nucleotide concentration tested. Note that the scale in ordenate is different in the two panels. Procedures are further detailed in Materials and Methods.

The difference of composition of mutant spectra generated during the replication of FMDV-wt and FMDV-3D(V173I), prompted us to compare nucleotide incorporation properties of the corresponding purified polymerases. To explore the enzymatic basis and to confirm the restriction of A → G and U → C transitions by FMDV-3D(V173I) in the presence of FU, the wt (3Dwt) and substituted 3D(V173I) polymerases were expressed, purified and studied biochemically and structurally. Both enzymes displayed similar binding to RNA (supplementary fig. S4, Supplementary Material online). Incorporation of standard nucleotides (NTPs) and of FUTP was quantified by determining *K*_d,NTP_ (dissociation of NTP from the elongation complex) and *k*_pol_ (constant for RNA elongation) using four sym/sub RNAs (Arnold and Cameron 2000; see fig. 2 andsupplementary fig. S5, Supplementary Material online, and table 3). While 3Dwt yielded comparable *k*_pol_/*K*_d,NTP_ for UTP and FUTP, 3D(V173I) displayed a drastically decreased incorporation of FUMP (*k*_pol_/*K*_d,NTP_ measures the catalytic efficiency of the reaction, taking into account the velocity of nucleotide incorporation and the affinity of the enzyme for the substrate). This gives a 3.2-fold higher selectivity (incorporation of UMP relative to FUMP) for the mutant than for the wt enzyme, contributed mainly by an increase in the dissociation of FU from the elongation complex resulting in a restriction of FUMP incorporation (fig. 2*B*;table 3). This result is in agreement with the reduction of transitions A → G and U → C observed in the mutant virus population compared with the wt when replicating in the presence of FU. In contrast, the two enzymes did not differ significantly regarding the relative incorporation of FU and C opposite G in the template and showed a strong preference for C incorporation (table 3; supplementary fig. S5, Supplementary Material online).

The incorporation of AMP and GMP opposite FU in the template indicated a 14-fold higher selectivity for A versus G as a result of substitution V173I, largely a consequence of increased dissociation of G relative to A (fig. 2*D*;table 3). Thus, the parameters of presteady state kinetics suggest that substitution V173I attenuates the mutagenic activity of FU by forcing recognition of the cognate nucleotide. Transitions G → A and C → U (which are the mutation types opposite to those favored by FU) will occur when FUMP is incorporated instead of CMP, directed by a G in the template. The kinetics parameters indicate that this misincorporation should not differ significantly between the two polymerases (supplementary fig. S5*C* and *D*, Supplementary Material online). However, for a FU in the template (originated by misincorporation opposite G) to give rise to a mutation it should direct the incorporation of A. 3D (V173I) exhibits higher selectivity than 3Dwt for the incorporation of A instead of G (fig. 2*C* and *D*;table 3). The end result is that 3D(V173I) will tend to generate a higher frequency of G → A transitions than 3D-wt thus compensating the tendency of FU to induce A → G and U → C mutations when replication is catalyzed by 3D-wt. Discrimination of nucleotides is greater when FU has to be incorporated rather than copied as template residue (table 3), suggesting that FU is more likely to induce mutations when it serves as template. Thus, the kinetic parameters determined with purified polymerase are consistent with the mutation repertoire quantified in the mutant spectra of FMDV-wt and FMDV 3D(V173I) passaged in presence of FU (table 2).

Substitution V173I did not affect the VPg uridylylation activity (supplementary fig. S6, Supplementary Material online), thereby establishing a structural and functional distinction between the mutagenic and inhibitory activities of FU.

### Structural Modifications of the Polymerase with Replacement V173I

**Fig. 3.—:**
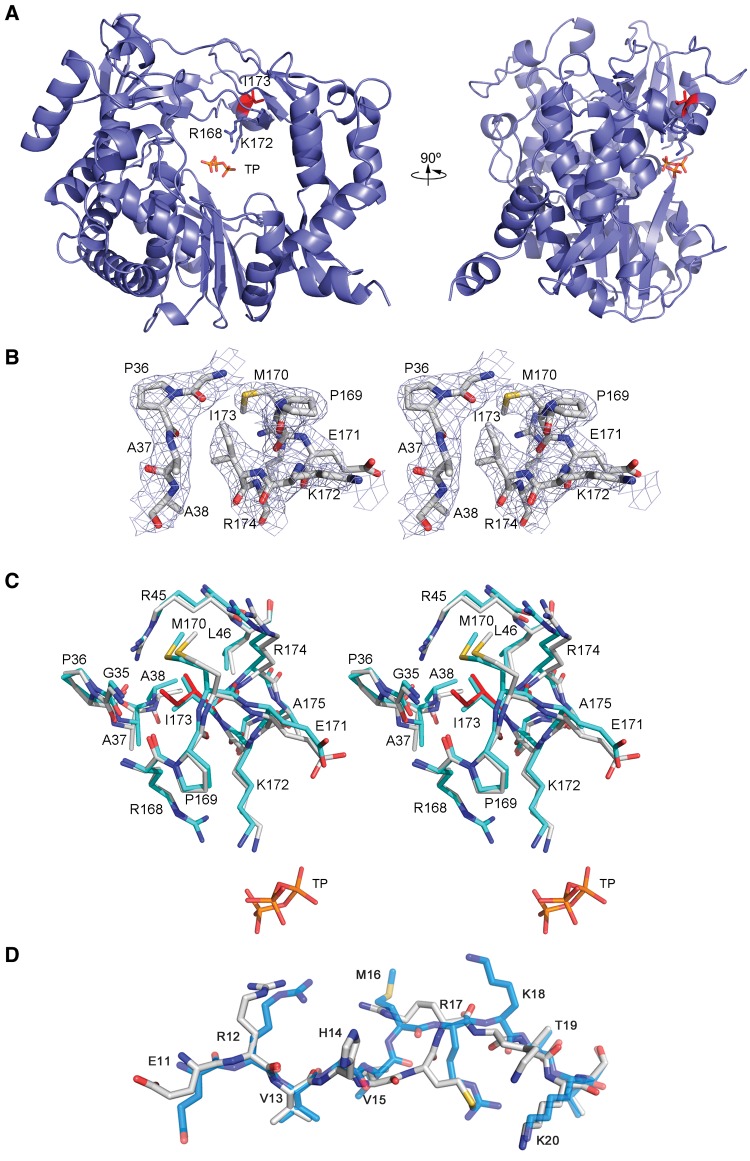
Structure of FMDV-3D(V173I). (*A*) The left and right panels show two views of 3D(V173I) protein rotated by 90°. The polymerase is depicted in blue ribbons with substituted amino acid I173 shown in sticks in red. The triphosphate moiety of the bound ATP and the motif F contacting side chains R168 and K172 are also shown as sticks and explicitly labeled. (*B*) Stereoview of σA-weighted |Fo| − |Fc| electron density map (contoured at 3σ) around the mutated residue I173. The substituted residues and surrounding amino acids were omitted from the phasing model. The model is placed inside in ball and stick representation and colored in atom type code. (*C*) Stereoview of the structural changes around the mutated I173 residue. 3D(V173I) polymerase is shown in white (with I173 depicted in red) and the superimposed 3Dwt in cyan (TP refers to the ATP triphosphate moiety). (*D*) Superimposition of the N-terminal region (residues E11–K20) of the FMDV-wt polymerase (cyan) and the FMDV-3D(V173I) polymerase (white). Information on data collection and refinement statistics is given in supplementary table S3, Supplementary Material online. The PDB accession codes for the 3Ds represented here are 1WNE for 3Dwt and 5DTN for 3D(V173I).

To investigate the structural modifications associated with the changes in nucleotide affinity, 3D(V173I) was crystallized and solved in two different crystal forms at 2.6 Å (P4_1_2_1_2 crystals) and 2.4 Å (P3_2_21 crystals) resolution, respectively (supplementary table S2, Supplementary Material online). The analysis of electron density maps allowed the tracing of the mutated and surrounding residues that were omitted from the initial model to eliminate model bias. The structure of 3D(V173I) was similar to 3Dwt (fig. 3). The superimpositions of all 476 amino acids of the two enzymes showed root mean square deviation (rmsd) values of 0.33 Å and 0.62 Å for space groups P4_1_2_1_2 and P3_2_21, respectively. Despite overall structural similarity, some significant changes were noted in the polymerase N-terminus (residues 14–18), and loop β9-α11 (residues 293–302, at the N-terminal side of polymerase motif B). In both space groups, the electron density indicates high flexibility around residues H14–K18 and this region could not be traced in the trigonal P3_2_21 crystals. In the P4_1_2_1_2 crystals the electron density was compatible with the tracing of two alternative conformations of the M16–R17 dipeptide main and side chains, with ∼50% occupancy each one. The first orientation was coincident with the position found for this region in the wt enzyme (Ferrer-Orta et al. 2004), with the side chain of R17 oriented towards the template channel, in a position accessible to the template RNA. In the second position, the main and side chains of residues M16 and R17 appeared reoriented, with the R17 side chain pointing to the polymerase interior, and interacting with residues N41 and Y285. Interestingly, this second orientation is coincident with that previously observed in the M296I, P44S, P169S (SSI) polymerase mutant, resistant to high concentrations of ribavirin (Agudo et al. 2010).

Two types of changes were observed in loop β9-α11 depending on the crystal type. While in the trigonal crystals the G299–S301 stretch was disordered and not visible in the electron density, the tetragonal crystals showed a 2.5 Å movement of G299 to S301 from their standard position in the wt enzyme, packed against the fingers (Ferrer-Orta et al. 2004), to a configuration where the loop protrudes towards the catalytic cavity in an orientation that would disturb the binding of the RNA template. Cocrystallization with sym/sub RNAs indicated that the bound RNA was disordered in the crystals, precluding a comparison with complexes with 3Dwt. A soaking of the crystals in 20 mM ATP resulted in the presence of the nucleotide bound to the NTP binding channel. However, only the triphosphate moiety of this incoming nucleotide was clearly seen in the electron density, interacting with the motif F residues R168 and K172, neighbor of the substituted residue I173 (fig. 3). Thus, localized structural changes are associated with altered nucleotide incorporation tendencies of 3D(V173I).

### Probing Targets of Selection with Predictive Algorithms and Experimental Determinations

**Fig. 4.—:**
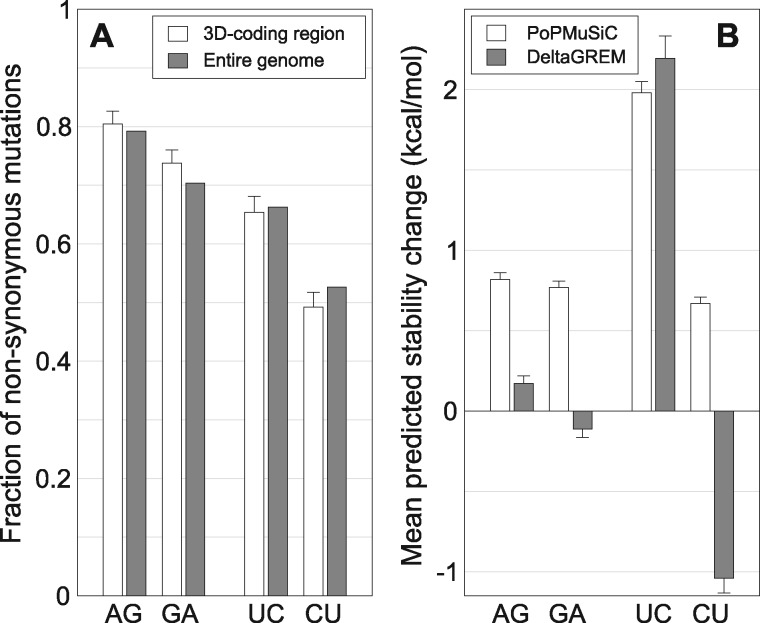
Predicted effect of transition mutations and non-synonymous mutations in the FMDV genome and 3D-coding region. (*A*) Fraction of non-synonymous mutations for each transition in the 3D-coding region and the entire FMDV genome; statistical significances are given in the text. (*B*) Mean change in folding free energy (ΔΔ*G*; negative values correspond to stabilizing substitutions) resulting from all possible non-synonymous transitions in the 3D-coding region, as predicted by PoPMuSiC and DeltaGREM. Both programs agree qualitatively on the comparison between different transitions, although DeltaGREM tends to predict more mutations as being stabilizing (ΔΔ*G* < 0). Procedures are detailed in Materials and Methods.

The biochemical and structural results prompted us to explore the possible consequences of the mutational effects on fitness at the molecular level. This objective has as difficulties that in RNA viruses both the RNA and the proteins are part of the phenotype, the genomic base composition can affect several steps in the virus life cycle, proteins are multifunctional, and selection may act on complex mutant spectra (Martin and Domingo 2008), and not necessarily on one or several dominant sequences [these points have been reviewed in Domingo 2016]. Since selection acts on phenotypes we sought to explore viral traits that might have driven the selection of FMDV with V173I in 3D upon virus replication in the presence of FU. According to the codon composition of the entire FMDV genome and of the 3D-coding region (Toja et al. 1999), the fraction of non-synonymous mutations is significantly larger for U → C than C → U mutations (Pearson’s chi-squared test: df = 1, *χ*^2^ = 64.0, *P* < 10^−14^), and for A → G than G → A mutations (Pearson’s chi-squared test: df = 1, *χ*^2^ = 36.9, *P* < 10^−8^; fig. 4*A*). Furthermore, the impact on 3D’s folding free energy (ΔΔ*G*) of every possible non-synonymous mutation was evaluated using PoPMuSiC and DeltaGREM (described in Materials and Methods). Both methods indicate that, on average, U → C mutations have a much stronger destabilizing effect on the protein than C → U mutations (Welch’s two-tailed *t*-test, for the comparison of the mean ΔΔ*G* as predicted by PoPMuSiC: df = 321, *t* = 17.5, *P* < 10^−15^). Similarly, A → G mutations are predicted to be on average more destabilizing than G → A mutations, although the differences are smaller (Welch’s two-tailed *t*-test, df = 521, *t* = 0.88, *P* = 0.38; fig. 4*B*). The relevance of the stability predictions is supported by evidence of stronger selection against mutations with a larger predicted destabilizing effect in the mutant spectra of FMDV and FMDV-3D(V173I; supplementary table S5, Supplementary Material online).

To further investigate the importance of protein stability as a target of selection, we expressed 10 mutants of the 3D polymerase and evaluated their melting temperatures (*T*_m_) via ThermoFluor experiments (table 4). Most mutants show a *T*_m_ decrease of at least 2 °C, three of them have a *T*_m_ lower than the temperature at which the passages were performed (37 °C), and three more were found to aggregate. In addition, we applied PoPMuSiC and DeltaGREM to estimate, for each mutant, the change in folding free energy at room temperature (ΔΔ*G*). An important reduction of the stability is predicted for 6 out of 10 mutants, by either or both computational methods (table 4). The genomes isolated from the mutant spectra are a surviving subset that eluded negative selection. Despite this bias, both the ThermoFluor experiments and the ΔΔ*G* predictions indicate that clones produced under FU-enhanced mutagenesis may present important alterations of 3D protein stability, with likely consequences for viral fitness. This result is in agreement with previous reports of decreasing fitness in subsets of genomes that remain viable in the process of ribavirin mutagenesis (Arias et al. 2013).

The average predicted RNA secondary structure stability was increased in the mutants displaying a larger content of the strongly interacting bases G and C (table 4 and supplementary fig. S7, Supplementary Material online). This effect is negligible for FMDV replicated in absence of FU (ΔΔ*G* = −0.03 kcal/mol), and strongest in presence of FU (ΔΔ*G* = −1.52 kcal/mol), which suggests the loss of a fine balance upon replication in the presence of FU. With FMDV-3D(V173I), the average RNA stability takes intermediate values, with no significant impact of the effect of FU.

**Table 3:** Kinetic Constants for Nucleotide Incorporation by FMDV 3Dwt and 3D(V173I)

RNA Substrate^a^	NTP^b^	3Dwt				3D(V173I)			
		*K*_d,NTP_ (µM)^c^	*k*_pol_ (s^−1^)^d^	*k*_pol_/*K*_d,NTP_ (µM^−1^ s^−1^)^e^	$\frac{\mathrm{(k}pol/K_{d},NTP)NTP}{\mathrm{(k}pol/K_{d},iNTP)iNTP}$^f^	*K*_d,NTP_ (µM)^c^	*k*_pol_ (s^−1^)^d^	*k*_pol_/*K*_d,NTP_ (µM^−1^ s^−1^)^e^	$\frac{\mathrm{(k}pol/K_{d},NTP)NTP}{\mathrm{(k}pol/K_{d},iNTP)iNTP}$^f^
Sym/sub-AU	A	44 ± 7	255 ± 44	5.8 ± 1.4	9.8 × 10^4^	60 ± 46	88 ± 14	1.5 ± 1.1	9.1 × 10^4^
	G	214 ± 71	12.6 ± 1.4 (× 10^−3^)	5.9 ± 2.1 (× 10^−5^)		635 ± 109	10.3 ± 0.8 (× 10^−3^)	1.6 ± 0.3 (× 10^−5^)	
Sym/sub-UA	U	54 ± 25	218 ± 27	4.0 ± 1.9	1.0	51 ± 9	196 ± 9	3.8 ± 0.7	3.2
	FU	36 ± 12	148 ± 8	4.1 ± 1.4		90 ± 36	108 ± 12	1.2 ± 0.5	
Sym/sub-UG	C	15 ± 6	307 ± 26	20.3 ± 8.9		20 ± 8	283 ± 23	14.4 ± 5.7	
	FU	24 ± 6	13.9 ± 0.7 (× 10^−3^)	5.8 ± 1.4 (× 10^−4^)	3.5 × 10^4^	30 ± 15	9.7 ± 1.1 (× 10^−3^)	3.3 ± 1.7 (× 10^−4^)	4.4 × 10^4^
	U	93 ± 38	9.7 ± 1.1 (× 10^−3^)	1.0 ± 0.4 (× 10^−4^)	2.0 × 10^5^	125 ± 20	4.2 ± 0.2 (× 10^−3^)	3.4 ± 0.6 (× 10^−5^)	4.2 × 10^5^
Sym/sub-FuG	A	273 ± 55	137 ± 10	0.50 ± 0.11	3.5 × 10^2^	29 ± 4	165 ± 6	5.69 ± 0.81	49.0 × 10^2^
	G	107 ± 28	0.16 ± 0.01	1.45 ± 0.40 (× 10^−3^)		130 ± 31	0.15 ± 0.01	1.16 ± 0.29 (× 10^−3^)	

a Symmetrical substrates (sym/subs) used in the assays depicted in fig. 2 and supplementary figure S5, Supplementary Material online.

b Nucleotidic substrate used as a triphosphate for incorporation at the second position of the sym/sub; the exception is sym/sub-FuG for which incorporation was measured at the first position.

c *K*_d,NTP_, dissociation constant for NTP binding.

d *k*_pol_, optimal polymerization rate constant.

e *k*_pol_/*K*_d,NTP_, catalytic efficiency.

f (*k*_pol_/*K*_d,NTP_)_NTP_/(*k*_pol_/*K*_d,iNTP_)_iNTP_, selectivity for discrimination in favor of incorporating the cognate nucleotide (NTP) instead of the incorrect nucleotide (iNTP).

Both PoPMuSiC and DeltaGREM concurred that, in absence of FU, the individual mutations in the mutant spectrum of FMDV-wt are less deleterious to protein stability than those found in the mutant spectrum of FMDV-3D(V173I) (e.g. with PoPMuSiC: mean ΔΔ*G* = 0.60 versus 1.09 kcal/mol; Welch’s two-tailed *t*-test: df = 59.2, *t* = 2.2, *P* = 0.03), while the opposite trend holds for mutations found in virus replicated in the presence of FU (supplementary fig. S7, Supplementary Material online). Besides the individual effect of each mutation, the increased mutation frequency resulting from FU mutagenesis markedly affects the average stability of 3D in each mutant spectrum. In consequence, according to DeltaGREM, the fraction of clones in which the stability of 3D is seriously altered (> 2.0 kcal/mol) increased from 4% for FMDV-wt replicated without FU to 32% with FU (Fisher’s test: *P* = 0.01), and from 12% to 23% for FMDV-3D(V173I) (Fisher’s test: *P* = 0.18). Similar results are obtained with PoPMuSiC (supplementary fig. S7, Supplementary Material online).

Interestingly, the average change of hydrophobicity is not significantly different from zero in the FMDV mutant spectrum passaged in the absence of FU (mean ΔH = −0.007; One-sample *t*-test (two-tailed): df = 27, *t* = −0.2, *P* = 0.85), despite the larger frequency of U → C mutations (supplementary fig. S7, Supplementary Material online). This suggests that increasing hydrophobicity may be less deleterious than decreasing it, and/or that compensating hydrophobicity changes may be profitable. The fine balance between mutations increasing and decreasing hydrophobicity is compromised in presence of FU, resulting in a significant loss of hydrophobicity (mean Δ*H* = −0.11; Welch’s two-tailed *t*-test: df = 54, *t* = 2.6, *P* = 0.01).

Thus, experimental determinations with mutant viral polymerases and predictive algorithms of the deleterious effects of amino acid substitutions suggest decreased functionality of the viral polymerase encoded by components of a mutant spectrum enriched in G and C residues.

## Discussion

The control of viral diseases using antiviral agents constitutes an important challenge. Lethal mutagenesis as an antiviral strategy arose as an application of error threshold concept included in quasispecies theory (Domingo and Schuster 2016b; Eigen and Schuster 1979; Perales and Domingo 2016; Schuster 2016; Tejero et al. 2016). The interest in applying lethal mutagenesis to combat viral disease was reinforced with the realization that some antiviral agents in use or in advanced clinical investigations such as ribavirin or favipiravir (T-705) may act as lethal mutagens against some viruses (Arias et al. 2014; Baranovich et al. 2013; Crotty et al. 2000; de Avila et al. 2016; Graci and Cameron 2006; Holland et al. 1990). Unfortunately the pertinacious adaptability of viral quasispecies has manifested also in the form of selection of viral mutants resistant to mutagenic nucleotide analogues (Agudo et al. 2010; Borderia et al. 2016; Ferrer-Orta et al. 2010; Pfeiffer and Kirkegaard 2005; Vignuzzi et al. 2006; Zeng et al. 2013).

The present study has addressed the selection of a FMDV mutant with amino acid substitution V173I in its polymerase that confers the virus a selective advantage during replication in the presence of FU. The selective advantage has been evidenced by the gradual increase in the frequency of amino acid substitution V173I in 3D when FMDV-RP was passaged in the presence of FU (fig. 1*B*), and the consistent FU concentration-dependent fitness increase of FMDV-3D(V173I) relative to FMDV-wt (figs. 1*C* and supplementary fig. S2, Supplementary Material online). Under the passage conditions of the fitness assays, neither reversion of V173I upon passage of FMDV-3D(V173I) in absence of FU, nor acquisition of V173I by FMDV-wt in the presence of FU, were observed (see Materials and Methods). The selective advantage of FMDV-3D(V173I) over FMDV-wt was associated with attenuation of the mutational bias in favor of A → G and U → C inflicted by FU upon FMDV. The increase of A → G and U → C transitions induced by FU was observed in previous experiments with FMDV-wt (Pariente et al. 2001; Sierra et al. 2000). The altered mutational preferences observed in the mutant spectra of FMDV-wt and FMDV-3D(V173I) are in agreement with the kinetics of nucleotide incorporation by the corresponding mutant polymerases. The mutant polymerase exhibits a compensatory increase of the mutation frequency from C to U in the presence of FU while maintaining a broad mutant spectrum in progeny virus, a hallmark of virus adaptability (Pfeiffer and Kirkegaard 2005; Vignuzzi et al. 2006). At the structural level (supplementary table S2, Supplementary Material online), the localized structural changes in 3D are similar to those previously found with other FMDV 3D mutants, which display decreased or increased incorporation of ribavirin–triphosphate (Agudo et al. 2010; Ferrer-Orta et al. 2010, 2007, 2015). These comparisons have revealed that very subtle structural adjustments may lead to either an increase or a decrease of nucleotide analogue incorporation during viral RNA synthesis.

The codon composition of an RNA viral genome is the result of a long evolutionary history, often in response to the host codon composition to ensure modulation of the rate of viral protein synthesis for virion production (Bosch et al. 2010; Mueller et al. 2010). Seeking to explore additional phenotypic traits that may have constituted a target for the selection of FMDV(V173I), we have applied some predictive and biochemical assays to document multiple effects at the RNA and protein level both in general for the FMDV genome, and for a sample of the mutants found in the relevant FU-mutagenized viral populations. It should be stressed that mutants detected among the molecular clones from mutagenized FMDV correspond to replication competent viruses, while the clones with the most deleterious mutations cannot be detected. Nevertheless, measurements with 10 mutant proteins revealed that many of them experienced substantial reductions of folding stability (table 4). Our predictive computations suggest that the effects of mutations on protein stability can be modulated by the mutation bias, in accordance with previous studies (Bastolla et al. 2006; Mendez et al. 2010). In the genetic background of FMDV, the mutations from U to C and from A to G, which are induced by FU, have a more deleterious effect than the mutations from C to U and from G to A, respectively. This is due to both a smaller fraction of synonymous mutations, and a greater impact of non-synonymous mutations on protein stability (figs. 4 and supplementary fig. S7, Supplementary Material online). These two effects may thus contribute to the fitness advantage displayed by FMDV-3D(V173I) in presence of FU, since the 3D(V173I) enzyme tends to diminish the frequency of G and C introduced in the genome (tables 2 and 3). The assumption that synonymous mutations are less likely to be deleterious is supported by the significantly larger than expected frequency of such mutations in the mutant spectra of the viral populations replicating in presence of FU, providing evidence of a stronger selection against non-synonymous mutations (table 2). Similar evidence indicates that the selection is even stronger against non-synonymous mutations with a large destabilizing impact (supplementary table S5, Supplementary Material online). Additional support for the validity of protein stability predictions used in the present study comes from a previous report of a significant correlation between PoPMuSiC predictions and protein fitness on a set of ∼1,000 mutants of β-lactamase TEM-1 (Jacquier et al. 2013).

The relationship between mutation bias and protein stability may be, at least in part, explained by the hydrophobicity of the different amino acid types (Bastolla et al. 2006; supplementary fig. S7, Supplementary Material online). Indeed, codons containing U at second position correspond to hydrophobic residues; on average, U → C mutations decrease hydrophobicity, while C → U mutations increase it. At the RNA level, U to C mutations tend to increase the secondary structure stability of the RNA, with possible influence on the stability of the viral genome and on protein expression (Plotkin and Kudla 2011).

Although protein stability is not the sole determinant of viral fitness, the predicted fractions of clones in which the stability of 3D is altered (supplementary fig. S7, Supplementary Material online) are qualitatively consistent with the comparative fitness assays (fig. 1*C* and supplementary fig. S2, Supplementary Material online); a quantitative investigation would require computational simulations based on more precise estimations of the mutational frequencies and biases. The complementary virological, enzymological, structural, and computational results presented here support the conclusion that the V173I substitution in 3D restores a required mutational balance, opening new perspectives on the role of mutational biases in viral and cellular evolution. For virology the results reinforce mutation modulation as a mechanism to escape lethal mutagenesis by purine and pyrimidine analogues, further illustrating the resources that RNA viruses have to confront hostile environments. For cell biology the results raise the intriguing possibility of external mutational activities affecting the replicative machinery over extended periods of evolution. These results support the view that mutation biases are under selective control, and they are consistent with the proposal, formulated in the context of microorganism evolution, that mutation biases can evolve through changes in the replicative machinery that are selected for their effects over extended periods of time (Mendez et al. 2010).

## Supplementary Material

Supplementary data are available at *Genome Biology and Evolution* online.

## Supplementary Material

Supplementary Data

## References

[evx075-B1] Agudo R, Arias A, Domingo E. 2009 5-Fluorouracil in lethal mutagenesis of foot-and-mouth disease virus. Future Med Chem. 1:529–539. 21426129 10.4155/fmc.09.26

[evx075-B2] Agudo R, 2008 Molecular characterization of a dual inhibitory and mutagenic activity of 5-fluorouridine triphosphate on viral RNA synthesis. Implications for lethal mutagenesis. J Mol Biol. 382:652–666. 18662697 10.1016/j.jmb.2008.07.033

[evx075-B3] Agudo R, de la Higuera I, Arias A, Grande-Perez A, Domingo E. 2016 Involvement of a joker mutation in a polymerase-independent lethal mutagenesis escape mechanism. Virology 494:257–266. 27136067 10.1016/j.virol.2016.04.023 PMC7111656

[evx075-B4] Agudo R, 2010 A multi-step process of viral adaptation to a mutagenic nucleoside analogue by modulation of transition types leads to extinction-escape. PLoS Pathog. 6:e1001072. 20865120 10.1371/journal.ppat.1001072 PMC2928812

[evx075-B5] Airaksinen A, Pariente N, Menendez-Arias L, Domingo E. 2003 Curing of foot-and-mouth disease virus from persistently infected cells by ribavirin involves enhanced mutagenesis. Virology 311:339–349. 12842623 10.1016/s0042-6822(03)00144-2

[evx075-B6] Andino R, Domingo E. 2015 Viral quasispecies. Virology 479-480:46–51. 25824477 10.1016/j.virol.2015.03.022 PMC4826558

[evx075-B7] Arenas M, Sanchez-Cobos A, Bastolla U. 2015 Maximum-likelihood phylogenetic inference with selection on protein folding stability. Mol Biol Evol. 32:2195–2207. 25837579 10.1093/molbev/msv085 PMC4833071

[evx075-B8] Arias A, 2005 Mutant viral polymerase in the transition of virus to error catastrophe identifies a critical site for RNA binding. J Mol Biol. 353:1021–1032. 16216271 10.1016/j.jmb.2005.09.022

[evx075-B9] Arias A, 2008 Determinants of RNA-dependent RNA polymerase (in)fidelity revealed by kinetic analysis of the polymerase encoded by a foot-and-mouth disease virus mutant with reduced sensitivity to ribavirin. J Virol. 82:12346–12355. 18829745 10.1128/JVI.01297-08 PMC2593321

[evx075-B10] Arias A, 2013 Molecular dissection of a viral quasispecies under mutagenic treatment: positive correlation between fitness loss and mutational load. J Gen Virol. 94:817–830. 23239576 10.1099/vir.0.049171-0

[evx075-B11] Arias A, Thorne L, Goodfellow I. 2014 Favipiravir elicits antiviral mutagenesis during virus replication in vivo. Elife 3:e03679. 25333492 10.7554/eLife.03679 PMC4204012

[evx075-B12] Arnold JJ, Cameron CE. 2000 Poliovirus RNA-dependent RNA polymerase (3D^pol^). Assembly of stable, elongation-competent complexes by using a symmetrical primer-template substrate (sym/sub). J Biol Chem. 275:5329–5336. 10681506 10.1074/jbc.275.8.5329

[evx075-B13] Baranovich T, 2013 T-705 (favipiravir) induces lethal mutagenesis in influenza A H1N1 viruses in vitro. J Virol. 87:3741–3751. 23325689 10.1128/JVI.02346-12 PMC3624194

[evx075-B14] Bastolla U. 2014 Detecting selection on protein stability through statistical mechanical models of folding and evolution. Biomolecules 4:291–314. 24970217 10.3390/biom4010291 PMC4030984

[evx075-B15] Bastolla U, Porto M, Roman HE, Vendruscolo M. 2005 Principal eigenvector of contact matrices and hydrophobicity profiles in proteins. Proteins 58:22–30. 15523667 10.1002/prot.20240

[evx075-B16] Bastolla U, Porto M, Roman HE, Vendruscolo M. 2006 A protein evolution model with independent sites that reproduces site-specific amino acid distributions from the Protein Data Bank. BMC Evol Biol. 6:43. 16737532 10.1186/1471-2148-6-43 PMC1570368

[evx075-B17] Bastolla U, Vendruscolo M, Knapp EW. 2000 A statistical mechanical method to optimize energy functions for protein folding. Proc Natl Acad Sci U S A. 97:3977–3981. 10760269 10.1073/pnas.97.8.3977 PMC18127

[evx075-B18] Borderia AV, Rozen-Gagnon K, Vignuzzi M. 2016 Fidelity variants and RNA quasispecies. Curr Top Microbiol Immunol. 392:303–322. 26499340 10.1007/82_2015_483 PMC7121553

[evx075-B19] Bosch A, Mueller S, Pintó RM. 2010 Codon biases and viral fitness In: Ehrenfeld E, Domingo E, Roos RP, editors. The picornaviruses. Washington, DC: ASM Press p. 271–283.

[evx075-B20] Castro C, Arnold JJ, Cameron CE. 2005 Incorporation fidelity of the viral RNA-dependent RNA polymerase: a kinetic, thermodynamic and structural perspective. Virus Res. 107:141–149. 15649560 10.1016/j.virusres.2004.11.004 PMC7125856

[evx075-B21] Crotty S, 2000 The broad-spectrum antiviral ribonucleoside ribavirin is an RNA virus mutagen. Nat Med. 6:1375–1379. 11100123 10.1038/82191

[evx075-B101] Charpentier N, Dávila M, Domingo E, Escarmís C. 1996 Long-term, large-population passage of aphthovirus can generate ad amplify defective noninterfering particles deleted in the leader protease gene. Virology. 23:10–8. 10.1006/viro.1996.0450 8806535

[evx075-B22] Dapp MJ, Patterson SE, Mansky LM. 2013 Back to the future: revisiting HIV-1 lethal mutagenesis. Trends Microbiol. 21:56–62. 23195922 10.1016/j.tim.2012.10.006 PMC3565075

[evx075-B23] de Avila AI, 2016 Lethal mutagenesis of hepatitis C virus induced by favipiravir. PLOS ONE. 11:e0164691. 27755573 10.1371/journal.pone.0164691 PMC5068784

[evx075-B24] Dehouck Y, 2009 Fast and accurate predictions of protein stability changes upon mutations using statistical potentials and neural networks: PoPMuSiC-2.0. Bioinformatics 25:2537–2543. 19654118 10.1093/bioinformatics/btp445

[evx075-B25] Dehouck Y, Kwasigroch JM, Gilis D, Rooman M. 2011 PoPMuSiC 2.1: a web server for the estimation of protein stability changes upon mutation and sequence optimality. BMC Bioinformatics. 12:151. 21569468 10.1186/1471-2105-12-151 PMC3113940

[evx075-B26] Derrida B. 1981 Random-energy model: An exactly solvable model of disordered systems. Phys Rev B. 24:2613–2626.

[evx075-B27] Domingo E. 2016 Virus as populations. Amsterdam: Academic Press, Elsevier.

[evx075-B28] Domingo E, editor. 2005 Virus entry into error catastrophe as a new antiviral strategy. Virus Res. 107:115–228.

[evx075-B29] Domingo E, Davila M, Ortin J. 1980 Nucleotide sequence heterogeneity of the RNA from a natural population of foot-and-mouth-disease virus. Gene 11:333–346. 6260578 10.1016/0378-1119(80)90073-6

[evx075-B30] Domingo E, Sabo D, Taniguchi T, Weissmann C. 1978 Nucleotide sequence heterogeneity of an RNA phage population. Cell 13:735–744. 657273 10.1016/0092-8674(78)90223-4

[evx075-B31] Domingo E, Schuster P. 2016a Quasispecies: from theory to experimental systems. Switzerland: Springer.

[evx075-B32] Domingo E, Schuster P. 2016b What is a quasispecies? Historical origins and current scope. Curr Top Microbiol Immunol. 392:1–22. 26318138 10.1007/82_2015_453

[evx075-B33] Domingo E, Sheldon J, Perales C. 2012 Viral quasispecies evolution. Microbiol Mol Biol Rev. 76:159–216. 22688811 10.1128/MMBR.05023-11 PMC3372249

[evx075-B34] Eigen M, Schuster P. 1979 The hypercycle. A principle of natural self-organization. Berlin: Springer. 10.1007/BF00450633 593400

[evx075-B35] Emsley P, Lohkamp B, Scott WG, Cowtan K. 2010 Features and development of Coot. Acta Crystallogr D Biol Crystallogr. 66:486–501. 20383002 10.1107/S0907444910007493 PMC2852313

[evx075-B36] Escarmís C, Dávila M, Domingo E. 1999 Multiple molecular pathways for fitness recovery of an RNA virus debilitated by operation of Muller's ratchet. J Mol Biol. 285:495–505. 9878424 10.1006/jmbi.1998.2366

[evx075-B37] Ferrer-Orta C, 2007 Sequential structures provide insights into the fidelity of RNA replication. Proc Natl Acad Sci U S A. 104:9463–9468. 17517631 10.1073/pnas.0700518104 PMC1890517

[evx075-B38] Ferrer-Orta C, 2004 Structure of foot-and-mouth disease virus RNA-dependent RNA polymerase and its complex with a template-primer RNA. J Biol Chem. 279:47212–47221. 15294895 10.1074/jbc.M405465200

[evx075-B39] Ferrer-Orta C, 2010 Structure of foot-and-mouth disease virus mutant polymerases with reduced sensitivity to ribavirin. J Virol. 84:6188–6199. 20392853 10.1128/JVI.02420-09 PMC2876637

[evx075-B40] Ferrer-Orta C, 2015 Multifunctionality of a picornavirus polymerase domain: nuclear localization signal and nucleotide recognition. J Virol. 89:6848–6859. 25903341 10.1128/JVI.03283-14 PMC4468482

[evx075-B100] García-Arriaza J, Manrubia SC, Toja M, Domingo E, Escarmís C. 2004 Evolutionary transition toward defective RNAs that are infectious by complementation. J Virol. 78(21):11678–11685. 15479809 10.1128/JVI.78.21.11678-11685.2004 PMC523252

[evx075-B41] Gohara DW, 2000 Poliovirus RNA-dependent RNA polymerase (3D^pol^). Structural, biochemical, and biological analysis of conserved structural motifs A and B. J Biol Chem. 275:25523–25532. 10827187 10.1074/jbc.M002671200

[evx075-B42] Graci JD, Cameron CE. 2006 Mechanisms of action of ribavirin against distinct viruses. Rev Med Virol. 16:37–48. 16287208 10.1002/rmv.483 PMC7169142

[evx075-B43] Graci JD, Cameron CE. 2008 Therapeutically targeting RNA viruses via lethal mutagenesis. Future Virol. 3:553–566. 19727424 10.2217/17460794.3.6.553 PMC2630198

[evx075-B44] Grande-Pérez A, Gómez-Mariano G, Lowenstein PR, Domingo E. 2005 Mutagenesis-induced, large fitness variations with an invariant arenavirus consensus genomic nucleotide sequence. J Virol. 79:10451–10459. 16051837 10.1128/JVI.79.16.10451-10459.2005 PMC1182645

[evx075-B45] Grande-Pérez A, Lázaro E, Lowenstein P, Domingo E, Manrubia SC. 2005 Suppression of viral infectivity through lethal defection. Proc Natl Acad Sci U S A. 102:4448–4452. 15767582 10.1073/pnas.0408871102 PMC555496

[evx075-B46] Grande-Pérez A, Sierra S, Castro MG, Domingo E, Lowenstein PR. 2002 Molecular indetermination in the transition to error catastrophe: systematic elimination of lymphocytic choriomeningitis virus through mutagenesis does not correlate linearly with large increases in mutant spectrum complexity. Proc Natl Acad Sci USA. 99:12938–12943. 12215495 10.1073/pnas.182426999 PMC130564

[evx075-B47] Holland JJ, Domingo E, de la Torre JC, Steinhauer DA. 1990 Mutation frequencies at defined single codon sites in vesicular stomatitis virus and poliovirus can be increased only slightly by chemical mutagenesis. J Virol. 64:3960–3962. 1695258 10.1128/jvi.64.8.3960-3962.1990 PMC249691

[evx075-B48] Jacquier H, 2013 Capturing the mutational landscape of the beta-lactamase TEM-1. Proc Natl Acad Sci U S A. 110:13067–13072. 23878237 10.1073/pnas.1215206110 PMC3740883

[evx075-B49] Kabsch W. 2010 XDS. Acta Crystallogr D Biol Crystallogr. 66:125–132. 20124692 10.1107/S0907444909047337 PMC2815665

[evx075-B50] Lauring AS, Frydman J, Andino R. 2013 The role of mutational robustness in RNA virus evolution. Nat Rev Microbiol. 11:327–336. 23524517 10.1038/nrmicro3003 PMC3981611

[evx075-B51] Loeb LA, 1999 Lethal mutagenesis of HIV with mutagenic nucleoside analogs. Proc Natl Acad Sci U S A. 96:1492–1497. 9990051 10.1073/pnas.96.4.1492 PMC15492

[evx075-B52] Markham NR, Zuker M. 2005 DINAMelt web server for nucleic acid melting prediction. Nucleic Acids Res. 33:W577–W581. 15980540 10.1093/nar/gki591 PMC1160267

[evx075-B53] Markham NR, Zuker M. 2008 UNAFold: software for nucleic acid folding and hybridization. Methods Mol Biol. 453:3–31. 18712296 10.1007/978-1-60327-429-6_1

[evx075-B54] Martin V, Domingo E. 2008 Influence of the mutant spectrum in viral evolution: focused selection of antigenic variants in a reconstructed viral quasispecies. Mol Biol Evol. 25:1544–1554. 18436553 10.1093/molbev/msn099

[evx075-B55] Mendez R, Fritsche M, Porto M, Bastolla U. 2010 Mutation bias favors protein folding stability in the evolution of small populations. PLoS Comput Biol. 6:e1000767. 20463869 10.1371/journal.pcbi.1000767 PMC2865504

[evx075-B56] Minning J, Porto M, Bastolla U. 2013 Detecting selection for negative design in proteins through an improved model of the misfolded state. Proteins 81:1102–1112. 23280507 10.1002/prot.24244

[evx075-B57] Mueller S, 2010 Live attenuated influenza virus vaccines by computer-aided rational design. Nat Biotechnol. 28:723–726. 20543832 10.1038/nbt.1636 PMC2902615

[evx075-B58] Murshudov GN, Vagin AA, Dodson EJ. 1997 Refinement of macromolecular structures by the maximum-likelihood method. Acta Crystallogr D Biol Crystallogr. 53:240–255. 15299926 10.1107/S0907444996012255

[evx075-B59] Pariente N, Sierra S, Lowenstein PR, Domingo E. 2001 Efficient virus extinction by combinations of a mutagen and antiviral inhibitors. J Virol. 75:9723–9730. 11559805 10.1128/JVI.75.20.9723-9730.2001 PMC114544

[evx075-B60] Perales C, Domingo E. 2016 Antiviral strategies based on lethal mutagenesis and error threshold. Curr Top Microbiol Immunol. 392:323–339. 26294225 10.1007/82_2015_459

[evx075-B61] Pfeiffer JK, Kirkegaard K. 2005 Increased fidelity reduces poliovirus fitness under selective pressure in mice. PLoS Pathogens. 1:102–110. 10.1371/journal.ppat.0010011 PMC1250929 16220146

[evx075-B62] Plotkin JB, Kudla G. 2011 Synonymous but not the same: the causes and consequences of codon bias. Nat Rev Genet. 12:32–42. 21102527 10.1038/nrg2899 PMC3074964

[evx075-B63] Potterton E, Briggs P, Turkenburg M, Dodson E. 2003 A graphical user interface to the CCP4 program suite. Acta Crystallogr D Biol Crystallogr. 59:1131–1137. 12832755 10.1107/s0907444903008126

[evx075-B64] Schuster P. 2016 Quasispecies on fitness landscapes. Curr Top Microbiol Immunol. 392:61–120. 26597856 10.1007/82_2015_469

[evx075-B65] Semisotnov GV, 1991 Study of the “molten globule” intermediate state in protein folding by a hydrophobic fluorescent probe. Biopolymers 31:119–128. 2025683 10.1002/bip.360310111

[evx075-B66] Sierra M, 2007 Foot-and-mouth disease virus mutant with decreased sensitivity to ribavirin: implications for error catastrophe. J Virol. 81:2012–2024. 17151116 10.1128/JVI.01606-06 PMC1797574

[evx075-B67] Sierra S, Dávila M, Lowenstein PR, Domingo E. 2000 Response of foot-and-mouth disease virus to increased mutagenesis. Influence of viral load and fitness in loss of infectivity. J Virol. 74:8316–8323. 10954530 10.1128/jvi.74.18.8316-8323.2000 PMC116341

[evx075-B68] Sobrino F, Dávila M, Ortín J, Domingo E. 1983 Multiple genetic variants arise in the course of replication of foot-and-mouth disease virus in cell culture. Virology 128:310–318. 6310859 10.1016/0042-6822(83)90258-1

[evx075-B69] Tejero H, Montero F, Nuno JC. 2016 Theories of lethal mutagenesis: from error catastrophe to lethal defection. Curr Top Microbiol Immunol. 392:161–179. 26210988 10.1007/82_2015_463

[evx075-B70] Toja M, Escarmis C, Domingo E. 1999 Genomic nucleotide sequence of a foot-and-mouth disease virus clone and its persistent derivatives. Implications for the evolution of viral quasispecies during a persistent infection. Virus Res. 64:161–171. 10518712 10.1016/s0168-1702(99)00089-1

[evx075-B71] Vignuzzi M, Stone JK, Arnold JJ, Cameron CE, Andino R. 2006 Quasispecies diversity determines pathogenesis through cooperative interactions in a viral population. Nature 439:344–348. 16327776 10.1038/nature04388 PMC1569948

[evx075-B72] Zeng J, 2013 An increased replication fidelity mutant of foot-and-mouth disease virus retains fitness in vitro and virulence in vivo. Antiviral Res. 100:1–7. 23880348 10.1016/j.antiviral.2013.07.008

