# Supplementary material for: Molecular and Functional Bases of Selection against a Mutation Bias in an RNA Virus
Source: Genome Biol Evol. 2017 May 1;9(5):1212–28. doi: 10.1093/gbe/evx075 (PMC5433387; doi:10.1093/gbe/evx075)
Supplement: Supplementary Data [file evx075_Supp.zip › Figures_ supp_22.03.17.pdf]

Figure S1

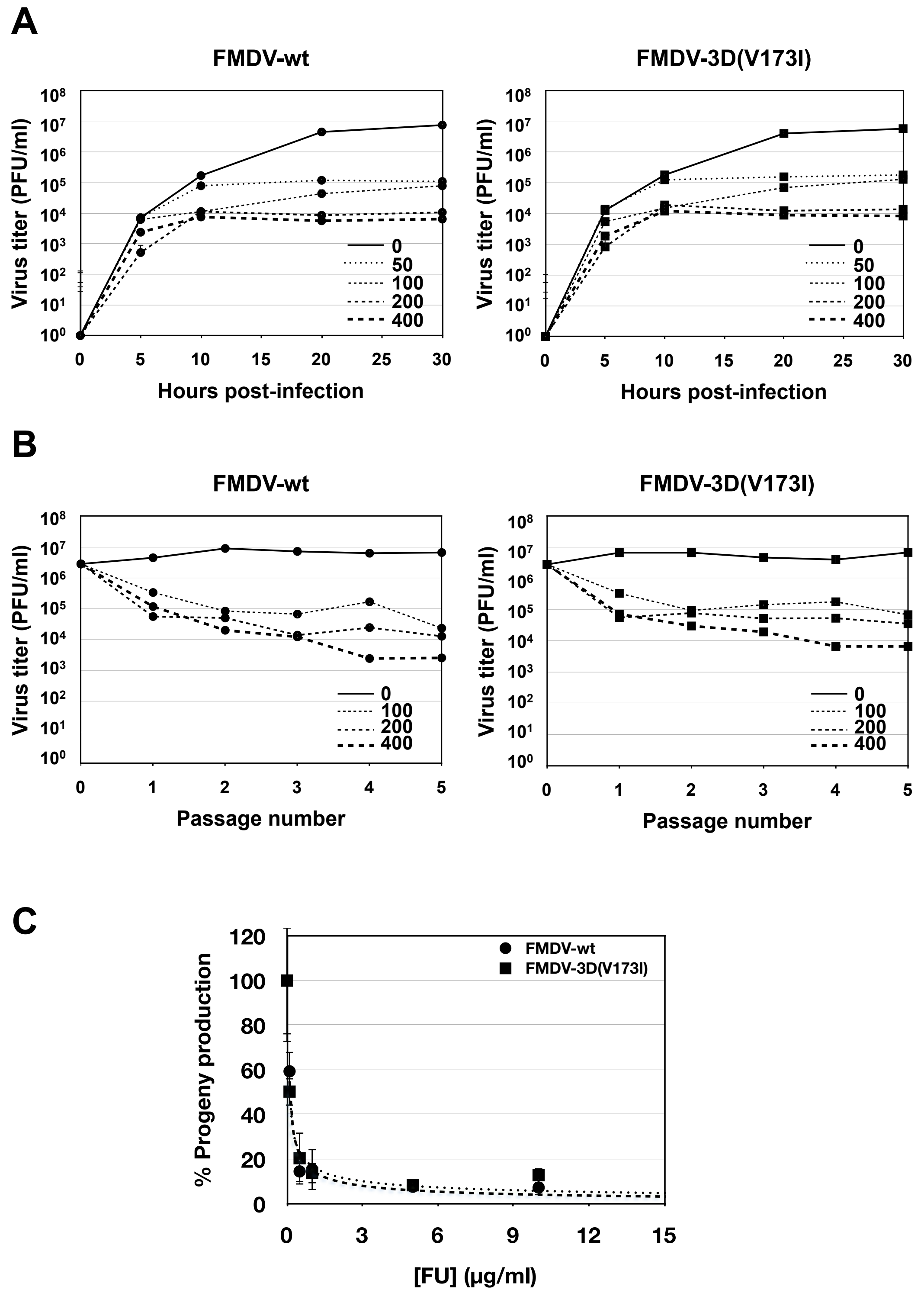
A
FMDV-wt
FMDV-3D(V173I)
Virus titer (PFU/ml)
Hours post-infection
0
50
100
200
400
B
FMDV-wt
FMDV-3D(V173I)
Virus titer (PFU/ml)
Passage number
0
100
200
400
C
% Progeny production
FMDV-wt
FMDV-3D(V173I)
[FU] (µg/ml)

Figure S2

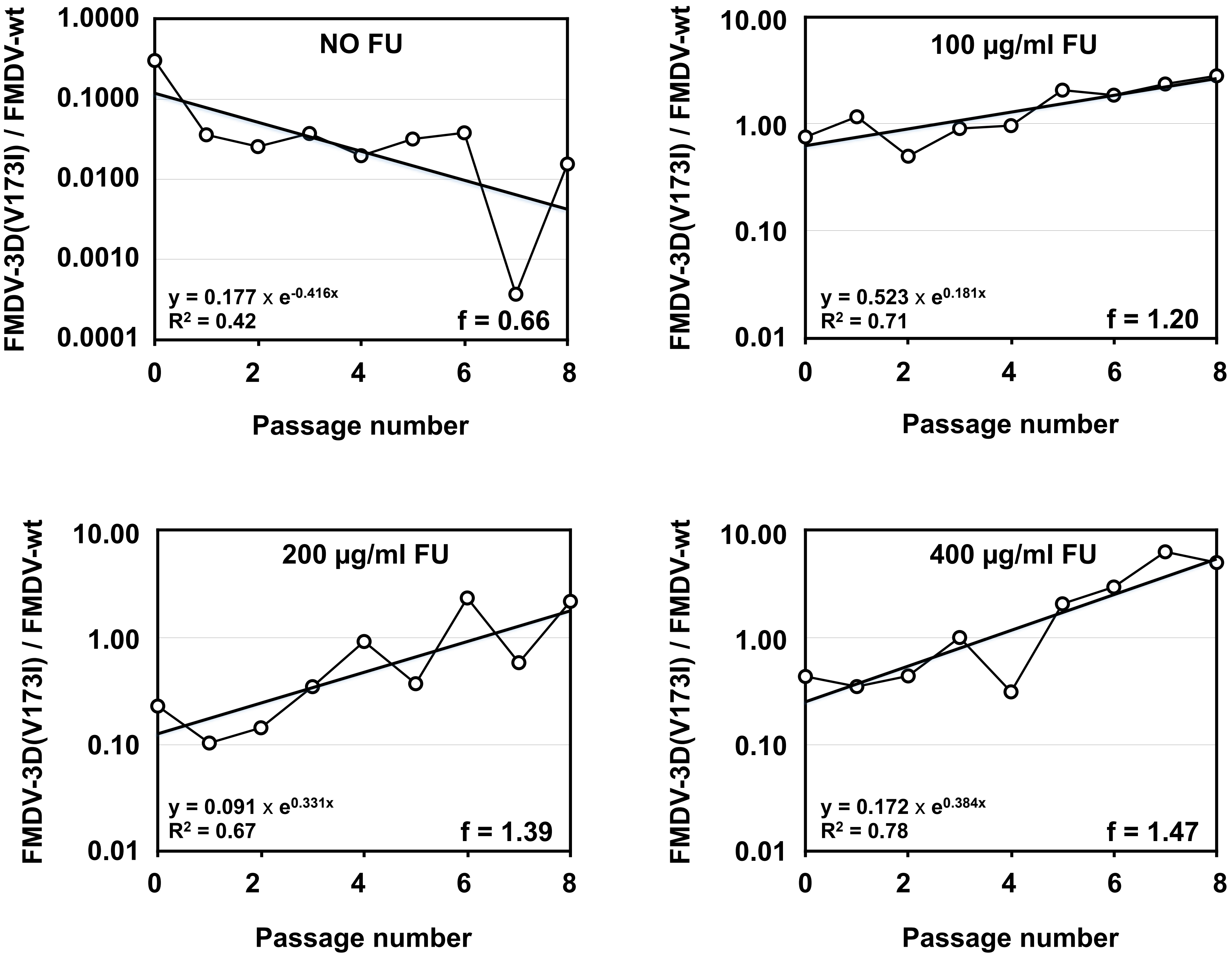
NO FU
FMDV-3D(V173I) / FMDV-wt
1.0000
0.1000
0.0100
0.0010
0.0001
y = 0.177 x e-0.416x
R2 = 0.42
f = 0.66
0
2
4
6
8
Passage number
100 µg/ml FU
10.00
1.00
0.10
0.01
y = 0.523 x e0.181x
R2 = 0.71
f = 1.20
200 µg/ml FU
y = 0.091 x e0.331x
R2 = 0.67
f = 1.39
400 µg/ml FU
y = 0.172 x e0.384x
R2 = 0.78
f = 1.47

Figure S3

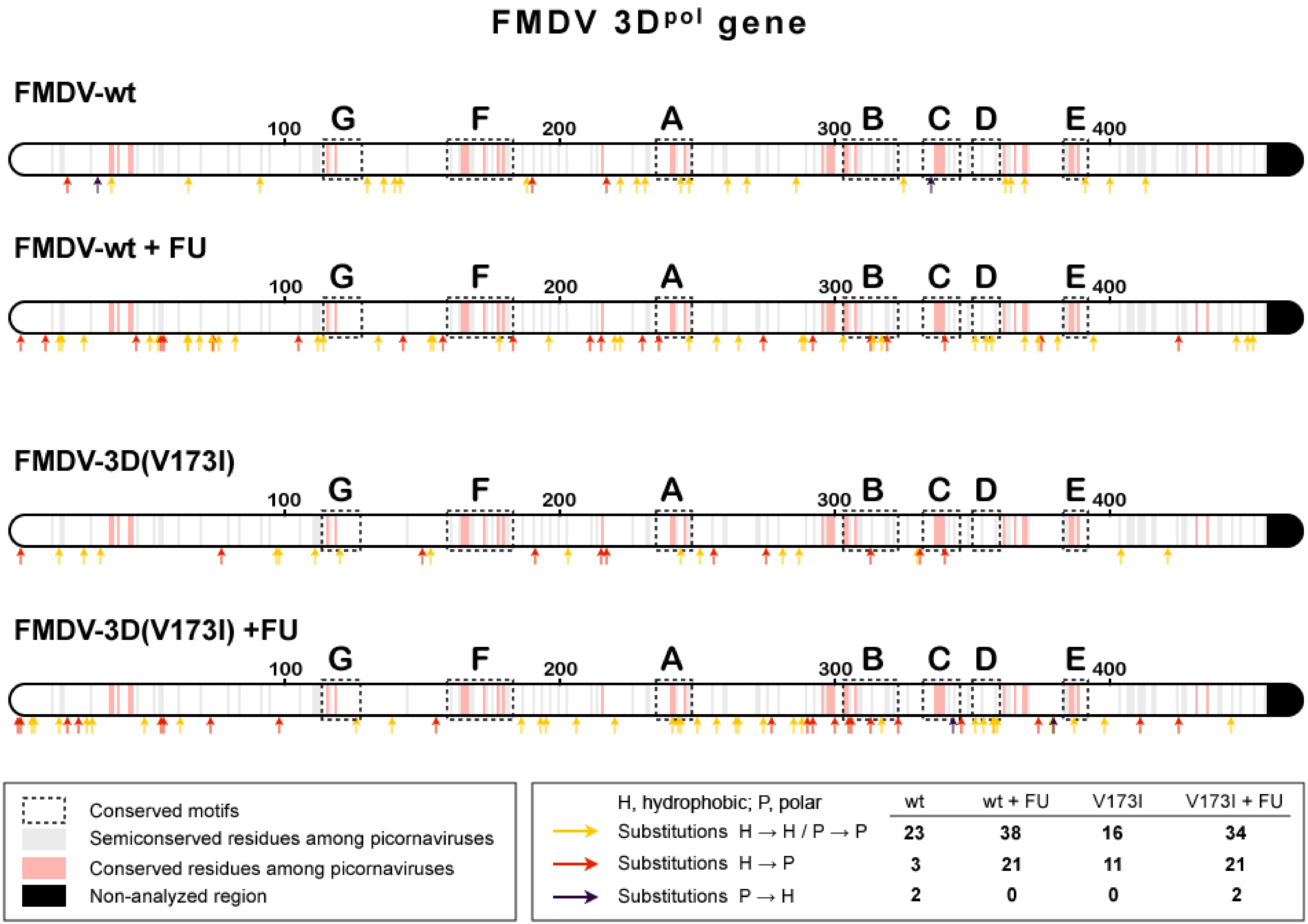
FMDV 3Dpol gene
FMDV-wt
FMDV-wt + FU
FMDV-3D(V173I)
FMDV-3D(V173I) +FU
G
F
A
B
C
D
E
100
200
300
400
Conserved motifs
Semiconserved residues among picornaviruses
Conserved residues among picornaviruses
Non-analyzed region
H, hydrophobic; P, polar
wt
wt + FU
V173I
V173I + FU
Substitutions H → H / P → P
23
38
16
34
Substitutions H → P
3
21
11
21
Substitutions P → H
2
0
0
2

Figure S4

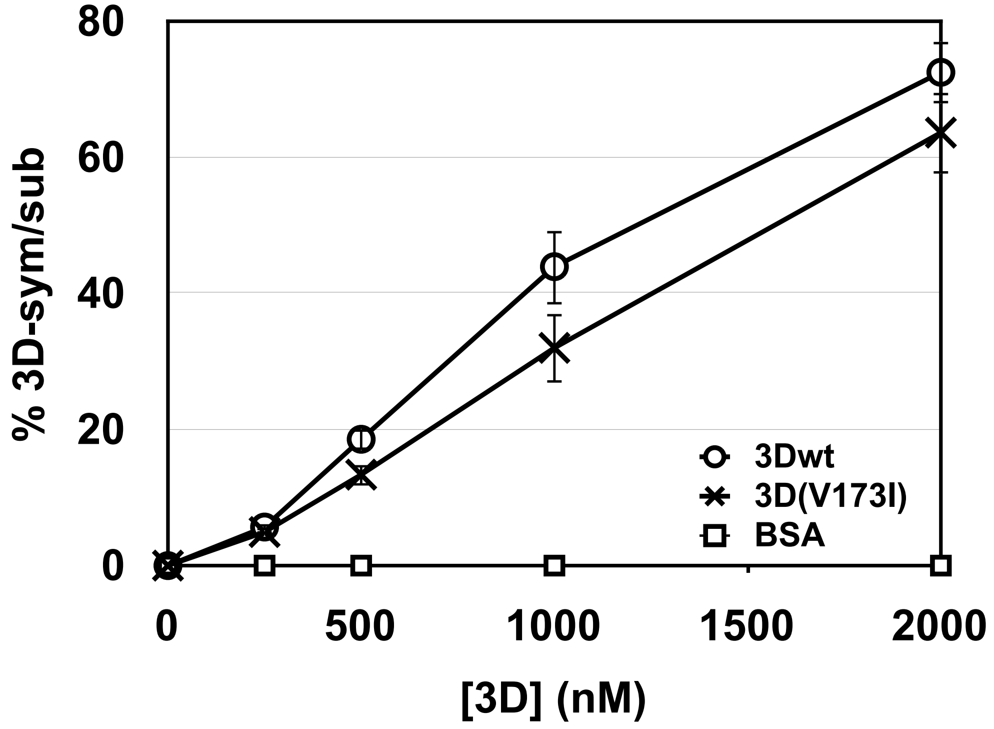
80
60
40
20
0
% 3D-sym/sub
0
500
1000
1500
2000
[3D] (nM)
3Dwt
3D(V173I)
BSA

Figure S5

A

sym/sub-AU

^{32}P-5‘ - **CGUAGGGCCC**
CCCGGGAUGC-5’-^{32}P

B

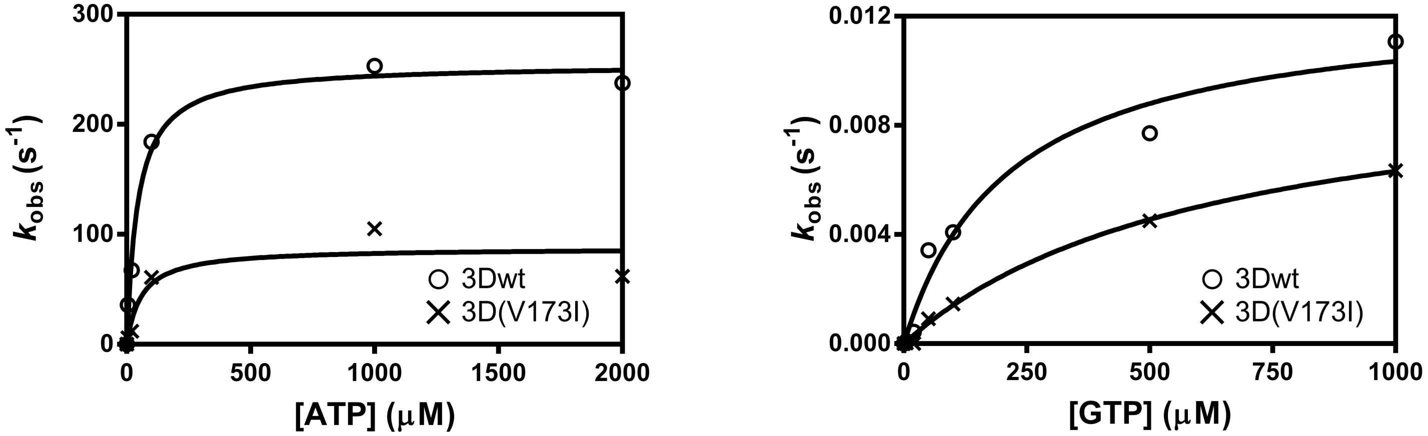

C

sym/sub-UG

^{32}P-5‘ - **ACGUGGGCCC**
CCCGGGUGCA-5’-^{32}P

D

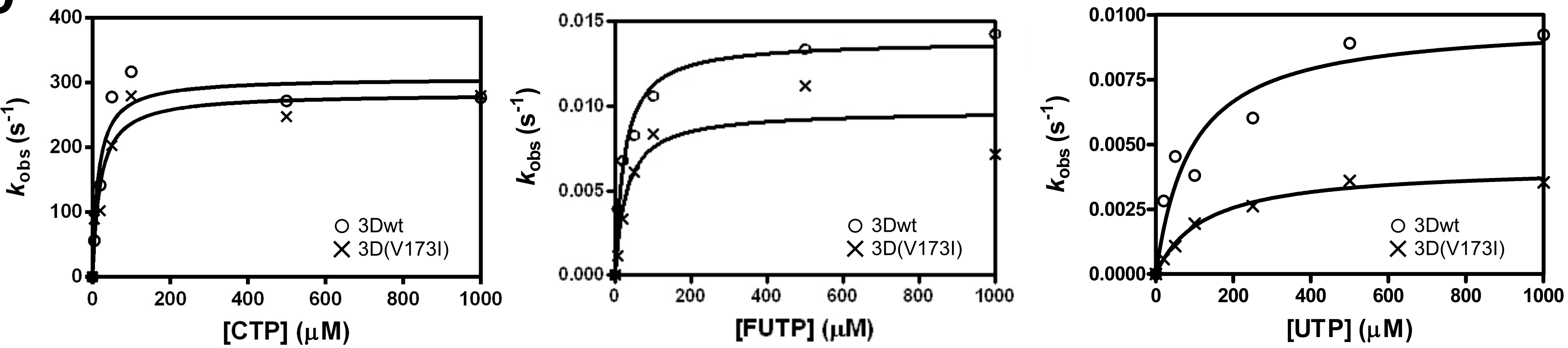

Figure S6

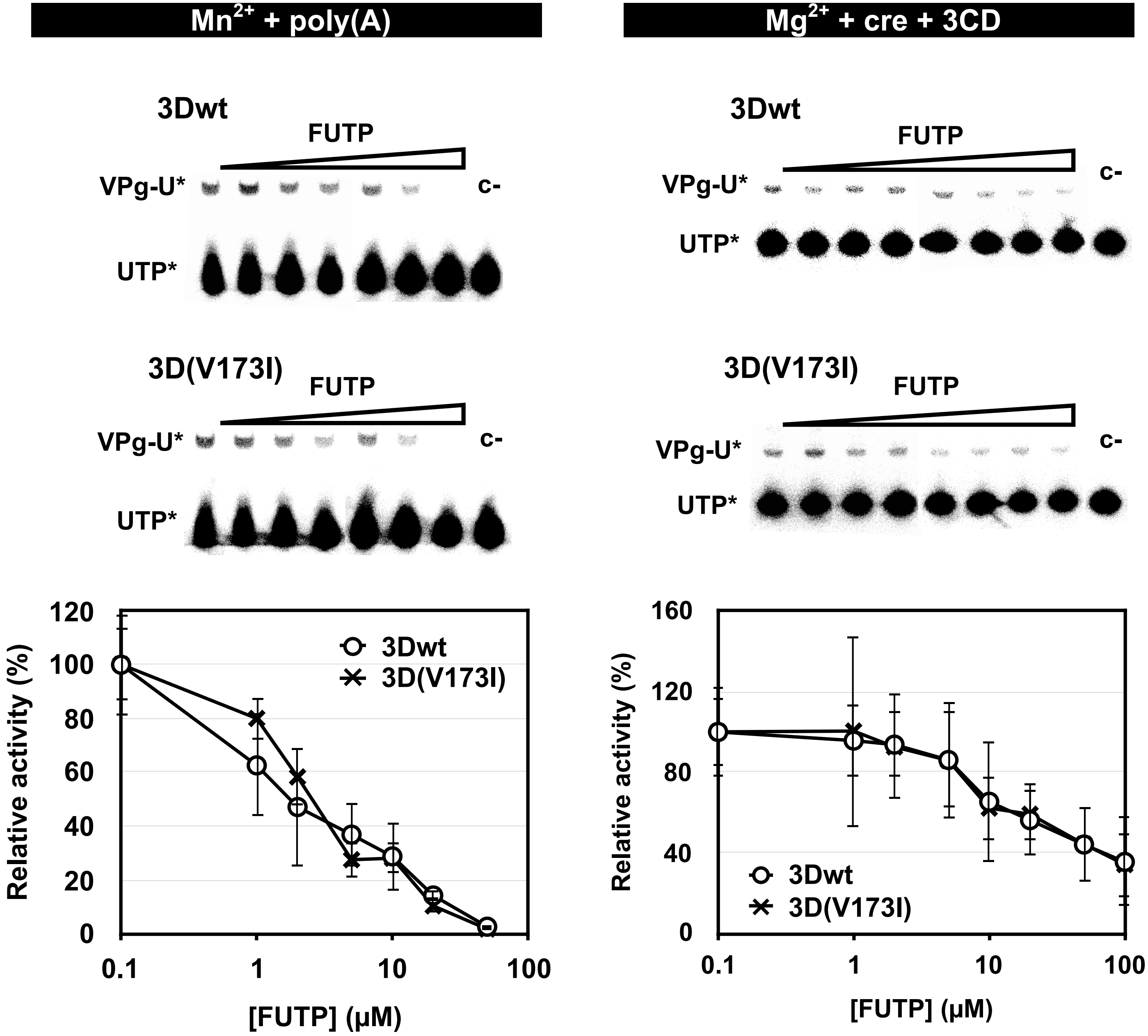

Mn2+ + poly(A)
Mg2+ + cre + 3CD
3Dwt
FUTP
VPg-U*
UTP*
c-
3D(V173I)
Relative activity (%)
[FUTP] (μM)
3Dwt
3D(V173I)
120
100
80
60
40
20
0
160
0.1
1
10
100

Figure S7

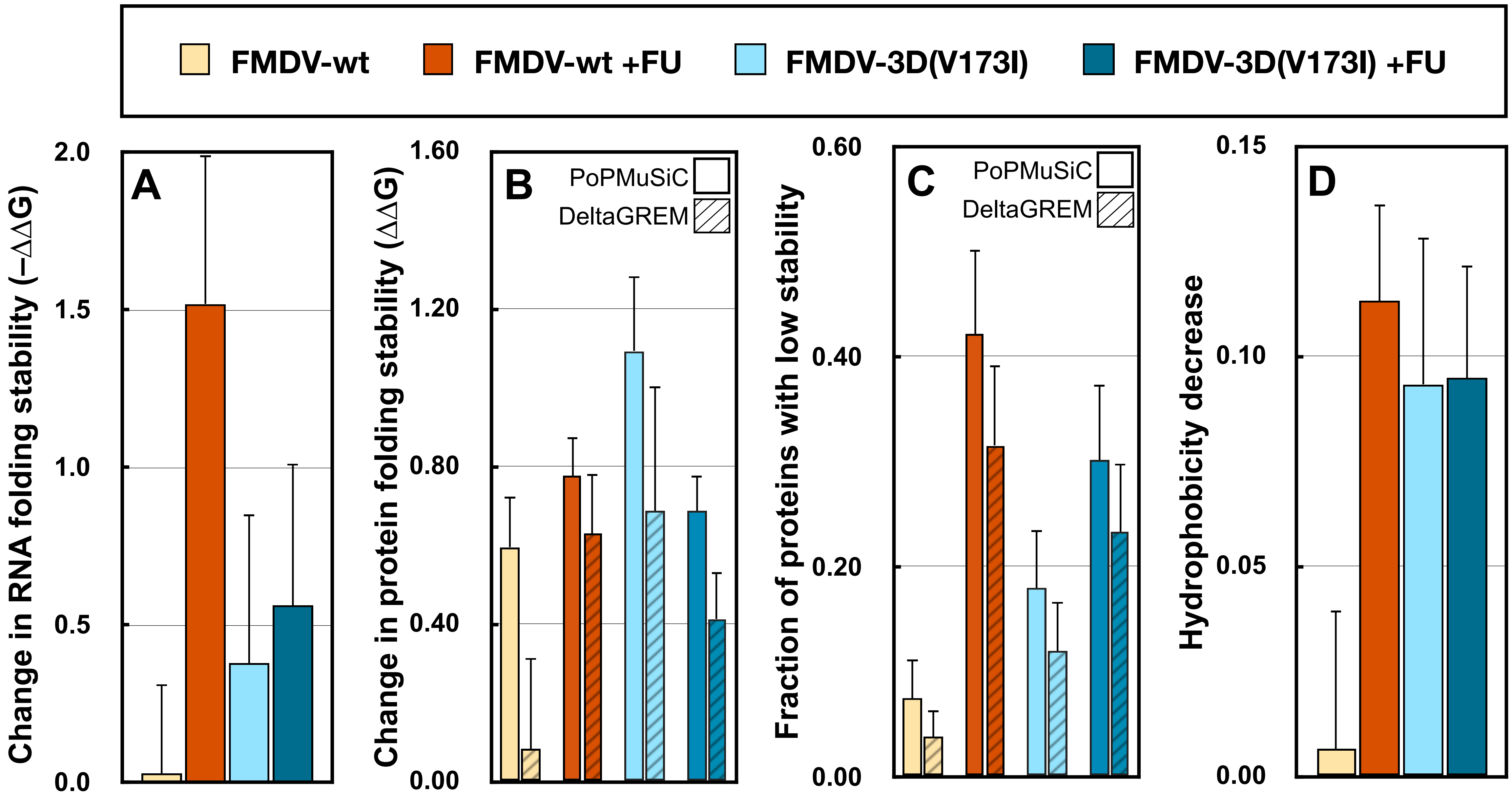

FMDV-wt
FMDV-wt +FU
FMDV-3D(V173I)
FMDV-3D(V173I) +FU
A
Change in RNA folding stability (−ΔΔG)
0.0
0.5
1.0
1.5
2.0
B
PoPMuSiC
DeltaGREM
Change in protein folding stability (ΔΔG)
0.00
0.40
0.80
1.20
1.60
C
PoPMuSiC
DeltaGREM
Fraction of proteins with low stability
0.00
0.20
0.40
0.60
D
Hydrophobicity decrease
0.00
0.05
0.10
0.15
