# Supplementary material for: Molecular and Functional Bases of Selection against a Mutation Bias in an RNA Virus
Source: Genome Biol Evol. 2017 May 1;9(5):1212–28. doi: 10.1093/gbe/evx075 (PMC5433387; doi:10.1093/gbe/evx075)
Supplement: Supplementary Data [file evx075_Supp.zip › Tables_supp_22.03.17.pdf]

Table S1. Oligonucleotides used to obtain and sequence the FMDV mutant polymerases.

Primer Name	Sequence (5'-3')[a]	Position[b]
**V173I plus**	CGCCCGATGGAGAAAATCCGTGCCGGC	7111
**V173I minus**	GCGAGTCTTGCCGGCACGGATTTTCTCCATCGGG	7146
**PolCKpnI**	GTTGG**T**AC**C**CACTCTGCTGGAGC	6502
**Pol1XbaI**	AAT**CTA**GATGTTTGGGGGATTATGCG	8041
**5'3D**	GGGTTGATCGTTGATACCAGAGA	6610
**AV2New**	TGTGGAAGTGTCTTTTGAGGAAAG	7783
**H27R plus**	CGTCGCGCGCGGTGTGTTCAATC	6681
**H27R minus**	GATTGAACACACCGCGCGCGACG	6703
**F74S plus**	CGCTGTCCCGCCGCTGCG	6824
**F74S minus**	CGCAGCGGCGGGACAGCG	6841
**V55A plus**	GTGTTGTCCTCGATGAAGCCATTTTCTCC	6755
**V55A minus**	GGAGAAAATGGCTTCATCGAGGACAACAC	6783
**F230S plus**	CGGCACACACTCCGCCCAATACAG	7287
**F230S minus**	CTGTATTGGGCGGAGTGTGTGCCG	7310
**D222G plus**	CAACCCTGATGTTGGTTGGCAGAGATTC	7260
**D222G minus**	GAATCTCTGCCAACCAACATCAGGGTTG	7287
**F374L plus**	CGACAAAGGTTTAGTTCTTGGTCACTCC	7719
**F374L minus**	GGAGTGACCAAGAACTAAACCTTTGTCG	7746
**L21P plus**	GCAAAACCAAGCCTGCACCCACCG	6659
**L21P minus**	CGGTGGGTGCAGGCTTGGTTTTGC	6682
**I56T plus**	GTCCTCGATGAAGTCACTTTCTCCAAGC	6760
**I56T minus**	GCTTGGAGAAAGTGACTTCATCGAGGAC	6787
**A243T plus**	GACGTGGACTATTCGACCTTTGATGC	7321
**A243T minus**	GCATCAAAGGTCGAATAGTCCACGTC	7346
**E264G plus**	GTTCCGCACGGGGTTCGGCTTC	7389
**E264G minus**	GAAGCCGAACCCCGTGCGGAAC	7410
**T228A plus**	GCAGAGATTCGGCGCACACTTCGC	7278
**T228A minus**	GCGAAGTGTGCGCCGAATCTCTGC	7301
**K369E plus**	CCATTACTCCAGCTGACGAAAGCGAC	7697
**K369E minus**	GTCGCTTTCGTCAGCTGGAGTAATGG	7722
**K18R plus**	GTCCATGTAATGCGCAGAACCAAGCTTG	6646
**K18R minus**	CAAGCTTGGTTCTGCGCATTACATGGAC	6673
**I196V plus**	CCAGGATGATGGTTGGCAGATTTTGTGC	7184
**I196V minus**	GCACAAAATCTGCCAACCATCATCCTGG	7211

[a]The underlined nucleotides indicate the residue at which a mutation is introduced. Primers PolCKpnI and Pol1XbaI (external to 3D), and 5′3D and AV2New (internal) were used to sequence the 3D-coding region. The letters in bold indicate residues that differ from the original FMDV sequence to be recognized by restriction enzymes KpnI and XbaI.

[b]According to C-S8c1 reference (Escarmís et al. 1999). Only 5' position is shown.

Table S2. Refinement statistics.

**Data collection**	$P4_12_12$ (PDB id. 5N95)	$P3_221$ (PDB id. 5N8X)
Resolution (Å)	46.3- 2.6 (2.7-2.6)	46.7-2.4 (2.5-2.40)
Space Group	$P4_12_12$	$P3_221$
Cell dimensions		
a, b, c (Å)	92.54 92.54 120.44	93.51 93.52 99.63
α, β, γ (º)	90 90 90	90 90 120
Rmerge	0.08 (0.76)	0.04 (0.43)
I/σI	12.2 (1.2)	22.0 (2.4)
Completeness (%)	99.2 (96.3)	99.4 (96.3)
Multiplicity	4.3 (3.9)	6.0 (3.6)
**Refinement**		
Resolution (Å)	46.3 -2.6	46.7-2.4
No. reflections (total/unique)	70448/ 16540	120085/20009
Rwork† / Rfree‡	23.7/27.8	24.5/28.0
No. Residues		
Protein	476	475
Ligand	1	
Water	44	32
Ions	-	1
B-factors (Å^2)		
Protein	48.1	74.9
Water + Ligands+Ions	64.0	60.3
R.m.s. deviations		
Bond lengths (Å)	0.007	0.008
Bond angles (°)	1.03	1.19
Ramachandran plot		
Residues in preferred regions	463/ 97.7%	451/ 95.3%
Residues in allowed regions	12/ 2.5%	21/ 4.4%

† Rwork = ∑hkl ||Fobs(hkl)| - |Fcalc(hkl)|| / ∑hkl |Fobs(hkl)|, where Fobs and Fcalc are the structure factors, deduced from measured intensities and calculated from the model, respectively.
‡ Rfree = as for Rwork but for 5% of the total reflections chosen at random and omitted from refinement.

Table S3: Nucleotide and amino acid changes in the mutant spectrum of foot-and-mouth disease virus populations passaged in the absence of 5-fluorouracil.

FMVD-wt p10[a]					FMDV-3D(V173I) p10[a]				
Mutation[b]	Amino acid substitution[b]	Δh[c]	PAM 250[d]	Clone[e]	Mutation[b]	Amino acid substitution[b]	Δh[c]	PAM 250[d]	Clone[e]
U6624C	-			30	U6620C	V4A	-0.272	0	50
U6671C	L21P	-0.423	-3	10	A6662G	K18R	0.046	3	32
C6704U	P32L	0.423	-3	29	A6689G	H27R	-0.019	2	26
G6718A	A37T	-0.078	1	25	G6706A	E33K	0.038	0	35
U6724C	-			50	U6838C	C77R	-0.238	-4	32
C6765U	-			42	A6898G	S97G	-0.003	1	28
A6771G	-			40	A6901G	I98V	-0.009	4	42
A6802G	K65E	-0.038	0	24	G6942A	M111I	0.243	2	31
U6843C	-			53	C6967U	P120S	-0.045	1	13
A6880G	T91A	0.078	1	8	U7002C	-			42
A6948G	-			28	U7058C	M150T	-0.116	-1	35
U6960C	-			14	A7066G	R153G	-0.083	-3	31
G6997A	A130R	-0.100	-2	32	U7167C	-			20
A7016G	N136S	-0.009	1	41	U7180C	Y191H	-0.262	0	30
G7027A	G140R	0.083	-3	31	A7216G	M203V	0.234	2	6
A7034G	E142G	0.002	0	5	C7227U	-			27
A7065G	-			5	U7253C	V215A	-0.272	0	43
A7172G	H188R	-0.019	2	17	C7254U	-			5
U7178G	L190R	-0.389	-3	24	U7258C	C217R	-0.238	-4	36, 42
U7258C	C217R	-0.238	-4	14	U7339A	F244I	0.010	1	43
U7272C	-			36	A7361G	D251G	0.077	1	35
A7274G	D222G	0.077	1	30	U7376C	M256T	-0.116	-1	32
A7284G	-			15	U7433C	L275P	-0.423	-3	10
A7291G	T228A	0.078	1	49	A7450G	T281A	0.078	1	32
G7300A	A231T	-0.078	1	33	A7455G	-			5
U7339C	F244L	0.017	2	2	A7468G	N287D	-0.089	2	42
A7349C	N247T	0.093	0	22	U7547C	I313T	-0.358	0	15
G7386A	-			51	U7569C	-			41

U7390C	F261L	0.017	2	30	A7597G	T330A	0.078	1	47
A7412G	H268R	-0.019	2	13, 18	U7600A	Y331N	-0.351	-2	50
A7468G	N287D	-0.089	2	53	U7628C	I340T	-0.358	0	27
U7509C	-			38	G7819A	A404T	-0.078	1	21
C7563U	-			41	A7871G	Q421R	0.004	1	43
G7583A	G325E	-0.002	0	15					
C7613U	S335F	0.451	-3	30					
A7695C	Q362H	0.022	3	35					
A7699G	I364V	-0.009	4	40					
A7714G	K369E	-0.038	0	49					
U7749C	-			14, 17					
A7781G	H391R	-0.019	2	26					
U7797C	-			47					
A7809C	K400N	-0.024	1	36					
U7846C	S413P	0.045	1	20					

Different mutations		43	Different mutations		33
Transitions (frequency x 10^{-4})[f]	A → G	16 (9.1)	Transitions (frequency x 10^{-4})[f]	A → G	12 (7.2)
	G → A	6 (3.2)		G → A	3 (1.7)
	U → C	13 (7.9)		U → C	13 (8.4)
	C → U	4 (2.0)		C → U	3 (1.6)
Transversions[g] (frequency x 10^{-4})[f]	A → C	3 (1.7)	Transversions[g] (frequency x 10^{-4})[f]	U → A	2 (1.3)
	U → G	1 (0.6)			
Synonymous (%)[h]		15 (35)	Synonymous (%)[h]		6 (18)
Non-synonymous (%)[i]		28 (65)	Non-synonymous (%)[i]		27 (82)
A→G U→C causing NS[j] (% among A→G U→C)		17 (61)	A→G U→C causing NS[j] (% among A→G U→C)		21 (78)
G→A C→U causing NS[j] (% among G→A C→U)		7 (25)	G→A C→U causing NS[j] (% among G→A C→U)		4 (15)
Repeated non-synonymous mutations (% among NS[j])		1 (4)	Repeated non-synonymous mutations (% among NS[j])		1 (4)

[a]The indicated FMDVs were subjected to 10 serial passages in BHK-21 cells.

[b]Mutations and deduced amino acid substitutions in the sequence of the 3D-coding region relative to the corresponding consensus sequence are indicated. Amino acid residues (single-letter code) are numbered from the N- to the C-terminus of 3D. Procedures for nucleotide sequencing are described in Materials and Methods.

[c]Δh represents the change of amino acidic hydrophobicity for each substitution. A positive value indicates a gain in hydrophobicity (changes towards non-polar amino acids) and a negative value indicates a loss in hydrophobicity (changes towards polar amino acids). Amino acid hydrophobicity is given in the legend for Fig.S3.

[d]PAM 250 represents the probability of occurrence of each substitution. According to the PAM 250 matrix, a positive value indicates higher probability than the expected from stochastic phenomena, and a negative value indicates lower probability than the expected from stochastic phenomena (Feng, D.F. & Doolittle, R.F. (1996). *Methods in Enzymol* **266**, 368-382).

[e]Clone identification number where the mutation was found.

[f]The frequency of occurrence of each mutation type is calculated as the number of mutations of that type (X to Y) divided by the total number of the corresponding base (X) (X to Y/ total X). In FMDV-wt, A→G and U→C transitions represent 67% of all different mutations observed in absence of FU, versus 84% in presence of FU (Table S4) ($p = 0.044$; Fisher's test). In FMDV-3D(V173I), A→G and U→C transitions represent 76% of all different mutations observed in absence of FU, versus 73% in presence of FU (Table S4) ($p = 0.82$; Fisher's test).

[g]The transversions not listed in the table were not found in the sequences analyzed.

[h]Synonymous: Number and percentage of mutations not leading to amino acid change (- in the second column).

[i]Non-synonymous: Number and percentage of mutations leading to amino acid change.

[j]NS is non-synonymous.

Table S4: Nucleotide and amino acid changes in the mutant spectrum of foot-and-mouth disease virus populations passaged in the presence of 5-fluorouracil.

FMDV-wt p10 + FU[a]					FMDV-3D(V173I) p10 + FU[a]				
Mutation[b]	Amino acid substitution[b]	Δh[c]	PAM 250[d]	Clone[e]	Mutation[b]	Amino acid substitution[b]	Δh[c]	PAM 250[d]	Clone[e]
U6613C	-			11	U6617C	I3T	-0.358	0	17
U6620C	V4A	-0.272	0	22, 30	U6620C	V4A	-0.272	0	34
U6624C	-			2	U6624C	-			29
U6647C	V13A	-0.272	0	17	A6632C	D8A	0.260	0	29
A6662G	K18R	0.046	3	1, 6, 9, 10, 13, 15, 16, 23, 27, 31	U6633C	-			36
A6664G	T19A	0.078	1	7	G6634A	V9M	-0.234	2	13
A6689G	H27R	-0.019	2	19	A6654G	-			37
G6714A	-			10	A6662G	K18R	0.046	3	35
U6717C	-			2	U6671C	L21P	-0.423	-3	23
A6741G	-			38	U6683C	V25A	-0.272	0	21
U6744C	-			25	G6692A	G28D	-0.077	1	11
U6746C	L46P	-0.423	-3	17	U6697C	F30L	0.017	2	36
G6760A	V51I	0.009	4	21	U6729C	-			32, 40
A6770G	E54G	0.002	0	35	G6735A	-			27
U6773C	V55A	-0.272	0	4, 36	U6744C	-			13, 16
U6776C	I56T	-0.358	0	14, 30	G6755A	G49D	-0.077	1	29
A6802G	K65E	-0.038	0	5	U6773C	V55A	-0.272	0	30
A6803G	K65R	0.046	3	28, 37	U6776C	I56T	-0.358	0	19, 23
A6815G	E69G	0.002	0	20	G6794A	G62E	-0.002	0	25
U6829C	F74L	0.017	2	31	U6827C	L73P	-0.423	-3	27
U6830C	F74S	-0.451	-3	19	A6858G	-			21
G6836A	R76H	0.019	2	23	U6902C	I98T	-0.358	0	12
U6843C	-			31	A6930G	-			15
G6853A	A82T	-0.078	1	11	C6933U	-			27
U6903C	-			16	G6972A	STOP			15
U6923C	V105A	-0.272	0	3	A6986G	K126R	0.046	3	17

A6930G	-			2	C7020U	-			14
A6944G	E112G	0.002	0	33	G7024A	V139I	0.009	4	2
G6949A	D114N	0.089	2	2	A7071G	-			35
C6951U	-			5	U7072C	Y155H	-0.262	0	31
U6966C	-			35	U7083C	-			15
C7005U	-			2	C7086U	-			13
U7009C	F134L	0.017	2	38	U7164C	-			19
A7029G	-			27	U7164G	-			18
U7037C	V143A	-0.272	0	8	G7165A	V186I	0.009	4	29
A7066G	R153G	-0.083	-3	23	A7172G	H188R	-0.019	2	29
A7068G	-			2	A7192G	M195V	0.234	2	25
A7070G	E154G	0.002	0	36	A7203G	-			11
U7079C	F157S	-0.451	-3	29	A7226G	N206S	-0.009	1	21
G7101A	-			33	G7236A	-			27
C7104U	-			20	U7257C	-			39
A7125G	-			7, 25	G7267A	D220N	0.089	2	22
A7128G	-			28, 30, 33, 37	U7275C	-			25, 42
A7141G	T178A	0.078	1	31	C7329U	-			21
U7157C	V183A	-0.272	0	20	A7331G	Y241C	-0.042	0	15
U7158C	-			32	G7336A	A243T	-0.078	1	41
A7195G	I196V	-0.009	4	9	U7339C	F244L	0.017	2	11
A7203G	-			17	C7350U	-			5
U7241C	I211T	-0.358	0	12	A7357G	S250G	-0.003	1	37
U7242C	-			35	U7378C	F257L	0.017	2	14, 15, 35
U7253C	V215A	-0.272	0	26	A7400G	E264G	0.002	0	41
A7268G	D220G	0.077	1	11	U7402C	F265L	0.017	2	17
A7274G	D222G	0.077	1	24	A7429G	I274V	-0.009	4	6, 33, 35
U7298C	F230S	-0.451	-3	4	A7438G	T277A	0.078	1	13
U7316C	V236A	-0.272	0	16	A7463G	Y285C	-0.042	0	42
A7348G	N247D	-0.089	2	5	A7472G	K288R	0.046	3	34
U7378C	F257L	0.017	2	18	U7478C	I290T	-0.358	0	27
U7402C	F265L	0.017	2	23, 31	C7479U	-			10

C7407U	-			18, 32	U7484C	V292A	-0.272	0	16
G7425A	-			35	U7507C	C300R	-0.238	-4	34
U7430C	I274T	-0.358	0	11	U7523C	I305T	-0.358	0	36
A7472G	K288R	0.046	3	11	U7526C	I306T	-0.358	0	8
G7475A	R289H	0.019	2	5	U7536C	-			34
U7482C	-			7	U7537C	-			3
U7484C	V292A	-0.272	0	28, 37	U7547C	I313T	-0.358	0	13
A7488G	-			2	A7559G	Y317C	-0.042	0	39
A7516G	T303A	0.078	1	25	A7572G	-			3, 28, 36
U7542C	-			4, 5	U7576C	Y323H	-0.262	0	19
U7547C	I313T	-0.358	0	15	U7617C	-			4
A7550G	Y314C	-0.042	0	17	C7626U	-			5
A7559G	Y317C	-0.042	0	3, 22	C7637U	A343V	0.272	0	37
U7564C	-			28	U7645C	Y346H	-0.262	0	36
U7565C	L319S	-0.468	-3	30, 37	U7647C	-			24
U7617C	-			26	U7650C	-			18
U7628C	I340T	-0.358	0	11	A7661G	E351G	0.002	0	34
U7650C	-			18, 32	A7670G	K354R	0.046	3	40
A7661G	E351G	0.002	0	30	G7671A	-			23, 39
C7672U	P355S	-0.045	1	14	A7681G	K358E	-0.038	0	16
C7677U	-			24	A7682G	K358R	0.046	3	15
U7678C	F357L	0.017	2	25	U7684C	S359P	0.045	1	27
A7683G	-			17, 20, 22, 29	C7692U	-			1
U7704C	-			31	C7698U	-			17, 33
A7715G	K369R	0.046	3	38	U7704C	-			13
A7725G	-			22	A7716G	-			14
U7728C	-			29	U7730C	F374S	-0.451	-3	21
U7729C	F374L	0.017	2	24	U7731C	-			43
U7733C	V375A	-0.272	0	22	U7734C	-			29
U7749C	-			1, 6, 10, 12, 13, 15, 16, 21, 22, 23, 27, 31,	U7744C	S379P	0.045	1	42

				33, 35, 38					
A7750G	T381A	0.078	1	6	C7745U	S379F	0.451	-3	22
A7790G	Y394C	-0.042	0	12	U7761C	-			29, 31
U7883C	I425T	-0.358	0	33	A7769G	K387R	0.046	3	38
C7887U	-			13	A7770G	-			12
A7896G	-			21	A7773G	-			12
U7945C	F446L	0.017	2	15	C7782U	-			23, 39
U7957C	F450L	0.017	2	36	U7788C	-			2, 11, 21, 32
A7963G	I452V	-0.009	4	20	U7801C	F398L	0.017	2	29
					U7839C	-			21
					U7841C	I411T	-0.358	0	27
					C7857U	-			35
					U7883C	I425T	-0.358	0	
					A7893G	-			38
					A7896G	-			25
					G7939A	E444K	0.038	0	35

Different mutations		96	Different mutations		103
Transitions (frequency x 10^{-4})[f]	A → G	35 (27.7)	Transitions (frequency x 10^{-4})[f]	A → G	29 (20.3)
	G → A	8 (5.9)		G → A	13 (8.5)
	U → C	46 (39.2)		U → C	46 (34.6)
	C → U	7 (4.9)		C → U	13 (8.1)
Transversions[g] (frequency x 10^{-4})[f]	A → C	-	Transversions[g] (frequency x 10^{-4})[f]	A → C	1 (0.7)
	U → G	-		U → G	1 (0.8)
Synonymous (%)[h]		37 (39)	Synonymous (%)[g]		45 (44)
Non-synonymous (%)[i]		59 (61)	Non-synonymous (%)[h]		57 (55)
Stop codons		0	Stop codons		1
A→G U→C causing NS[j] (% among A→G U→C)		53 (65)	A→G U→C causing NS[j] (% among A→G U→C)		45 (60)
G→A C→U causing NS[j] (% among G→A C→U)		6 (100)	G→A C→U causing NS[j] (% among G→A C→U)		11 (42)
Repeated non-synonymous mutations (% among NS[j])		9 (15)	Repeated non-synonymous mutations (% among NS[j])		3 (5)

[a]The indicated FMDVs were subjected to 10 passages in BHK-21 cells in presence of FU (400µg/ml).

[b]Mutation and deduced amino acid substitutions in the sequence of the 3D-coding region relative to the consensus sequence are indicated. Amino acid residues (single-letter code) are numbered from the N- to the C-terminus of 3D. The statistical significance of the difference between the number of mutations in the populations compared is given in the legend of Table 2 of the main text. Procedures for nucleotide sequencing are described in Materials and

Methods.

[c]Δh represents the change of amino acidic hydrophobicity for each substitution. A positive value indicates a gain in hydrophobicity (changes towards non-polar amino acids) and a negative value indicates a loss in hydrophobicity (changes towards polar amino acids).

[d]PAM 250 represents the probability of occurrence of each substitution. According to the PAM 250 matrix, a positive value indicates higher probability than the expected from stochastic phenomena, and a negative value indicates lower probability than the expected from stochastic phenomena (Feng, D.F. & Doolittle, R.F. (1996) *Methods in Enzymol* **266**, 368-382).

[e]Clone identification number where the mutation was found.

[f]The frequency of occurrence of each mutation type is calculated as the number of mutations of that type (X to Y) divided by the total number of the corresponding base (X) (X to Y/ total X). In FMDV-wt, A→G and U→C transitions represent 67% of all different mutations observed in absence of FU, versus 84% in presence of FU (Table S3) ($p = 0.044$; Fisher's test). In FMDV-3D(V173I), A→G and U→C transitions represent 76% of all different mutations observed in absence of FU, versus 73% in presence of FU (Table S3) ($p = 0.82$; Fisher's test).

[g]The transversions not listed in the table were not found in the sequences analyzed.

[h]Synonymous: Number and percentage of mutations not leading to amino acid change (- in the second column).

[i]Non-synonymous: Number and percentage of mutations leading to amino acid change.

[j]NS is non-synonymous.

Table S5

Evidence of selection against substitutions with larger (predicted) destabilizing effect on 3D.

	Viral population[a]	Number of substitutions[b]	Mean ΔΔG (expected, kcal/mol)[c]	Mean ΔΔG (observed, kcal/mol)[d]	p-value (one-tailed t-test)
Unique mutations	FMDV-wt	28	1.03	**0.91**	0.25
	FMDV-3D(V173I)	27	1.29	1.34	-
	FMDV-wt + FU	59	1.38	**1.28**	0.20
	FMDV-3D(V173I) + FU	57	1.36	**1.23**	0.13
	All samples merged	171	1.30	**1.21**	0.10
All mutations	FMDV-wt	29	1.03	**0.91**	0.23
	FMDV-3D(V173I)	28	1.31	1.36	-
	FMDV-wt + FU	76	1.35	**1.15***	0.04
	FMDV-3D(V173I) + FU	62	1.37	**1.26**	0.17
	All samples merged	195	1.30	**1.18***	0.03

[a] Wild-type FMDV or mutant FMDV-3D(V173I) were subjected to 10 serial passages in the absence or presence of FU (400 µg/ml) (+FU).

[b] Number of different substitutions found in the mutant spectrum (Unique mutations) (top rows), or the total number of substitutions found in the mutant spectrum (All mutations) (bottom rows).

[c] Mean change in folding free energy expected from individual amino acid substitutions in each mutant spectrum, as predicted by PoPMuSiC. To obtain these values, each substitution observed in the sample was assigned the average stability change predicted for all possible non-synonymous mutations of the same type in the 3D-coding region (see fig. 4B).

[d] Mean change in folding free energy resulting from the individual amino acid substitutions observed in each mutant spectrum, as predicted by PoPMuSiC. Values in bold indicate that the mean observed ΔΔG is smaller than the mean expected ΔΔG.
